# Polymer types ingested by northern fulmars (*Fulmarus glacialis*) and southern hemisphere relatives

**DOI:** 10.1007/s11356-020-10540-6

**Published:** 2020-08-26

**Authors:** Susanne Kühn, Albert van Oyen, Elisa L. Bravo Rebolledo, Amalie V. Ask, Jan Andries van Franeker

**Affiliations:** 1 Wageningen Marine Research, Ankerpark 27, 1781 Den Helder, AG Netherlands; 2 CARAT GmbH, Harderhook 22, 46395 Bocholt, Germany; 3 Bureau Waardenburg BV, Varkensmarkt 9, 4101 Culemborg, CK Netherlands; 4 Norwegian Polar Institute, Fram Centre, P.O. 6606 Langnes, N-9296 Tromsø, Norway

**Keywords:** Marine plastic debris, Ingestion, Procellariiformes, Northern fulmar (*Fulmarus glacialis*), Near infrared spectroscopy (NIR), Fourier transform infrared spectroscopy (FTIR)

## Abstract

Although ingestion of plastic by tubenosed seabirds has been documented regularly, identification of the polymer composition of these plastics has rarely been described. Polymer assessment may assist in identifying sources and may indicate risks from additives occurring in specific types of polymers. Using known test materials, two identification methods Fourier transform infrared spectroscopy and near infrared spectroscopy (FTIR and NIR) were compared. Although both methods were found to be similarly suitable for identification of plastic polymers, a significant difference was observed in identification of natural materials. FTIR frequently misclassified natural materials as being a synthetic polymer. Within our results, an 80% match score threshold functioned best to distinguish between natural items and synthetics. Using NIR, the historical variability of plastics ingested by northern fulmars (*Fulmarus glacialis*) from the Dutch sector of the North Sea was analysed for three time periods since the 1980s. For the more recent decade, variability between fulmars from different regions in the northeast Atlantic was investigated. Regional variation was further explored by analysing plastics obtained from the stomachs of southern hemisphere relatives of the fulmar (southern fulmar, cape petrel, snow petrel) and Wilson’s storm petrel. Results show that proportional abundance of polymer types in these seabirds is closely related to the plastic categories that they ingest (e.g. pellets, foam, fragments). The uptake of different plastic categories and related polymer types most likely reflects spatial and temporal variations in availability rather than ingestion preferences of the birds.

**Electronic supplementary material:**

The online version of this article (10.1007/s11356-020-10540-6) contains supplementary material, which is available to authorized users.

## Introduction

Tubenosed seabirds (Procellariiformes) are known to ingest debris including plastics from the sea surface. At a global scale, 91 of 144 known procellariform seabird species have been recorded to ingest plastic (Kühn and van Franeker 2020). In many cases, ingestion may occur intentionally, but usually for unknown reasons. Resemblance to prey is often suggested, but Ryan (1987) linked plastic ingestion especially to seabird species with a less specialized diet. Ingestion of plastic might also occur accidentally. For example, albatrosses forage on strings of eggs of flying fish that are attached to pieces of floating plastic (Pettit et al. 1981). Finally, plastic ingestion will partly occur indirectly, e.g. by foraging on prey that ingested plastics itself (Hipfner et al. 2017). The effects of plastic on marine wildlife are largely unknown due to the many factors that might influence the level of harm. These factors include uptake, retention time, the digestion mode of different organisms and in particular the broad variety of polymers, shape and chemical burden of the plastics themselves. The retention time of plastic is difficult to determine in free-ranging seabirds. It depends on the shape and the size of the ingested plastic in relation to body size, the wearing process in the stomach and a threshold size that is needed to excrete the plastic (Ryan 2015). One study indicates a loss of 75% of the plastic load per month for fulmarine petrels (Van Franeker and Law 2015), while others suggest a complete loss of plastics in less than 2 months (Terepocki et al. 2017) or up to many months for a broader variety of species (Ryan and Jackson 1987; Ryan 2015).

Ingested plastics may cause mechanical disruptions and a false feeling of satiation (Kühn et al. 2015). Chemical additives added during the manufacturing process of different polymers and substances adsorbed from the marine environment are of concern, as potential toxic substances may harm marine organisms (Oehlmann et al. 2009; Teuten et al. 2009; Rochman 2015; Tanaka et al. 2015). Polymer assessments of plastics ingested by marine wildlife may be of value in relation to toxicity of specific polymers, their degradation products or specific additives used (Lithner et al. 2011).

Plastic ingestion by seabirds has been recorded from the 1960s onwards (Threlfall 1968; Kenyon and Kridler 1969), and an increasing body of publications proves that plastic and the ingestion of it by marine megafauna occur over all the world’s oceans (Provencher et al. 2017). In recent years, research quantifying the abundance of plastics in organisms (frequency of occurrence, average number and sometimes average mass of plastic items) has been complemented by investigations of polymer types and related chemical burdens (Tanaka et al. 2013; Tanaka et al. 2019; Rizzi et al. 2019; Nelms et al. 2019; Avio et al. 2020). This development is related to the technical progress in analytical methods such as infrared and mass spectroscopy and to the focus on small-sized plastics that require advanced identification techniques. Studies investigating plastic ingestion by fish are relatively recent and frequently use Fourier transform infrared spectroscopy (FTIR) or Raman spectroscopy to identify polymer types (e.g. Löder and Gerdts 2015; Lusher et al. 2013; Rummel et al. 2016; Pellini et al. 2018; Wieczorek et al. 2018; Kühn et al. 2020). Several earlier studies (e.g. Yamashita et al. 2011; Amélineau et al. 2016; Avery-Gomm et al. 2016; Pham et al. 2017; Van Franeker et al. 2018; Tanaka et al. 2019; Rizzi et al. 2019) provided some information, but on the larger scale, the identification of plastic polymers in marine megafauna is still relatively scarce. Data on the composition of polymer types is needed to evaluate potential toxic consequences of plastic ingestion because different plastic types contain different types of additives, leaching behaviour and degradation products (Lithner et al. 2011). Spectroscopy produces light reflection or transmission spectra that can be compared with a library of known polymer spectra. The match between spectra is often expressed in percentages; however, the threshold of acceptance of the results, as being reliable, differs among studies.

Plastic ingestion by northern fulmars (*Fulmarus glacialis*) has been observed since the 1970s (Bourne 1976; Furness 1985; Van Franeker 1985). From 2002 onwards, this procellariform seabird has been used as a monitoring tool for marine debris for the Oslo-Paris Convention for the Protection of the Marine Environment of the North-East Atlantic (OSPAR) in the North Sea. The fulmar is one of the most studied species with regard to the quantification of plastic pollution and has been proven to be a suitable monitoring tool to assess changes in abundance and types of plastic (Van Franeker et al. 2011; Van Franeker and Law 2015; OSPAR 2017; OSPAR 2019). Although single birds may undertake impressive foraging trips, 10 out of 12 fulmars tracked on trips lasting 4 to 15 days stayed within 100 km distance of the colony (Edwards et al. 2013). Similarly, the individual site foraging fidelity in northern gannets (*Morus bassanus*) tends to be within a scale of tens of kilometres (Wakefield et al. 2015), and behavioural traits suggest that wintering distributions show similar characteristics with birds staying within restricted areas known to them (Piper 2011). Boreal fulmars do not show seasonal migration patterns (Mallory et al. 2012). As a consequence of foraging site fidelity, the average amount of plastics in fulmar stomachs of larger sample sizes will reflect pollution patterns over restricted spatial scales over longer periods of time (Van Franeker et al. 2011). Research has been expanded from the Netherlands to the entire North Sea area and further to the North Atlantic, including the Faroe Islands (Van Franeker et al. 2011), Iceland (Kühn and Van Franeker 2012) and Svalbard (Trevail et al. 2015). Based on a common standardized protocol (Van Franeker 2004; Van Franeker et al. 2011; OSPAR 2015), these results are easily comparable with other studies and study regions, such as the Canadian (sub)Arctic (Mallory 2006; Mallory 2008; Provencher et al. 2009; Avery-Gomm et al. 2018) and the North Pacific Ocean (Donnelly-Greenan et al. 2014; Nevins et al. 2005; Avery-Gomm et al. 2012; Terepocki et al. 2017). The outcomes of these studies report the frequency of occurrence, average number of pieces and plastic mass. From these studies, it appears that ingested quantities of plastic by fulmars tend to decrease with increasing latitude (Mallory 2008; Van Franeker et al. 2011; Kühn and Van Franeker 2012; Baak et al. 2020). Recently, in fulmars from the Dutch coast, a decreasing trend in the mass of ingested plastic has been observed (Van Franeker and Kühn 2019).

NIR was used to evaluate potential differences in ingested polymers on a temporal and spatial scale. We used archived samples from long-term fulmar studies to analyse the polymer composition of ingested plastics from different time periods in the North Sea. To compare regional differences, the North Sea results were compared with the polymer composition of plastics from fulmars from other locations in the northeast Atlantic Ocean (Faroe Islands, Iceland and Svalbard) and to plastics obtained from a number of related species in the Southern Ocean. Temporal and spatial variation of plastic ingestion can be useful to detect changes in the composition of plastics and to monitor the effectiveness of certain mitigation measures. For example, Van Franeker and Law (2015) demonstrated that industrial plastic pellets have decreased in ocean gyres and in northern fulmars and linked this to the successful implementation of regulations to avoid loss of pellets during production and transportation. Comparable findings were made by Vliestra and Parga (2002) in the North Pacific Ocean and by Ryan (2008) in the South Atlantic Ocean. Seabirds are therefore seen as suitable sentinels to monitor changes in plastic pollution.

The starting point of our study aimed at identifying a suitable threshold level for the reliability of our near infrared spectroscopy (NIR) results in comparison with FTIR analyses for the same set of known polymer and natural particles. The second aim was to use NIR results to deepen the knowledge of plastics ingested by northern fulmars and related seabird species from the Southern Ocean.

## Methods

### IR method evaluation

FTIR analysis has been more regularly used than NIR for polymer identification of ingested plastics. For the current study however, NIR was available to analyse plastics from petrel stomachs. To detect potential differences in both techniques, a comparative experiment was prepared, applying NIR and FTIR on a large selection of known synthetics and natural items. Different threshold values for the reliability of the results were used. For each of these results, NIR and FTIR were compared to see whether the two methods delivered comparable and reliable results.

A total of 200 test items were prepared for both NIR and FTIR polymer assessments. These test items reflected a broad variety of items potentially encountered in stomachs of marine organisms, both natural prey (remains) and marine debris.

A total of 117 items were man-made materials, and 83 items were of natural origin (Table 1). Plastics covered a wide range of colours and plastic categories (24 raw industrial pellets and 66 consumer-type particles of categories as identified by Van Franeker et al. (2011), such as sheets, threads, foams and fragments). In this system, fibres from clothing were not considered. The details of each test item can be found in the Online Supplement Table 1.

**Table 1:** Numbers of man-made and natural items tested with FTIR and NIR, respectively

Category	Sub-category	*n* items
Man-made items (*n* = 117)	Synthetic polymers (22 different polymer types)	103
	Compostable bioplastics	8
	Balloon rubber	2
	Paraffin/palmfat	4
Natural items (*n* = 83)	Fish bones and otoliths, eye lenses of squid and fish, crab carapaces and shells of bivalves and insects, skin, bill structures and feathers of different seabirds, gastropods. Natural non-food items such as stones, wood, wool, seaweed and seeds	83

In infrared spectroscopy, results of polymer identification are usually associated with a percentage match score. This score indicates the degree of overlap between the sample spectrum and the spectrum of the most similar substance from the IR library. For this study, the reliability of infrared spectroscopy plastic identification was tested at the thresholds of 70%, 80% and 90%. All items were analysed with both NIR (DTS-PHAZIR-1624 for 1600–2400 nm library details: Online Supplement Table 2) and FTIR (ATR-FTIR; Shimadzu Prestige 21, 10 Scans, Libraries: ATR Polymer 2, IRs Polymer 2, T-Polymer).

Results in either method were categorized as:

1. Correctly identified: for synthetic polymer test items above selected percentage and material correctly categorized; for natural items below selected percentage irrespective of chosen library substance.
2. Falsely identified: above selected percentage; but incorrectly categorized; for both synthetic polymers and natural items.
3. Not identified: for synthetic polymers below required percentage; note that natural materials with a match score below the chosen reliability percentage were considered as correctly classified, i.e. not being a known synthetic polymer. Spectra for natural items are often not available in the IR libraries. Therefore, a score lower than the intended match score was accepted as correct identification, meaning their identification as polymer was not successful.

Polymers identified by FTIR as HDPE and LDPE were grouped as PE, as the NIR library did not allow that distinction.

### Seabird samples

For this study, we analysed plastics ingested by northern fulmars (*Fulmarus glacialis*) and three of its Southern Ocean close relatives (southern fulmar *Fulmarus glacialoides*, cape petrel *Daption capense* and snow petrel *Pagodroma nivea*) plus the Wilson’s storm petrel (*Oceanites oceanicus*). For the temporal analysis of plastics from beached fulmars in the Netherlands, a selection of 129 birds covering three decennia (1980–2011) was used. For a regional comparison between fulmars from the North Atlantic, stomach contents of northern fulmars from the Netherlands were compared with samples from the Faroe Islands, Iceland and Svalbard. Samples from Antarctica were collected during research expeditions between 1984 and 1998 in the Windmill Islands area near the Australian Casey station (66° S, 110° E) in eastern Antarctica. Overall, we used plastics from stomachs of 317 individual birds, as specified in Table 2.

**Table 2:** Seabird species, location, year and sample number (n) of examined procellariform samples

Species	Country	Years	*n* birds
Northern fulmar	Netherlands	1982–2019	129
*Northern fulmar*	*NL1980-89*	*1980–1989*	*(58)*
*Northern fulmar*	*NL1909-99*	*1990–1999*	*(32)*
*Northern fulmar*	*NL2010-11*	*2010–2019*	*(39)*
Northern fulmar	Faroe Islands	2011	50
Northern fulmar	Iceland	2011	46
Northern fulmar	Svalbard	2013	35
Northern fulmar	Total	1982–2019	**260**
Wilsons storm petrel	Antarctica	1986–1998	45
Cape petrel	Antarctica	1985–1986	9
S. fulmar	Antarctica	1984	2
Snow petrel	Antarctica	1985	1
Antarctica	Total	1984–1998	**57**

Italics were used for sub categories (e.g. Netherlands per decennia). Bold was used for summarizing data

### Dissection protocol

All birds were dissected according to guidelines by Van Franeker (2004) and OSPAR (2015). In short, external measurements (head, tarsus and wing length and bill length and depth) and details on moult (primary and secondary moult, down score) were recorded. Internally, the sex, age, organ health and condition were assessed, and the stomachs were removed. The stomach content was sieved on 1 mm mesh size, and all hard items were sorted under a stereomicroscope as either natural food or plastics. The plastic items were further split into plastic categories according to Van Franeker et al. (2011). Industrial pellets are small, often cylindric-shaped plastic granules, around 4 mm in diameter, and the raw material is used for the production of plastic products. Microbeads are much smaller, often spherical plastic granules usually 1 mm or less in diameter, which became known because they were used in many types of cosmetics but are actually used in many industrial applications. User plastics include sheets (e.g. plastic bags, agricultural foil), threads (ropes, nets, fishing line, etc.), foams (e.g. foamed polystyrene or polyurethane) and fragments (rigid items often broken of larger plastic objects). Plastic items such as balloons, cigarette filters and rubber are included in the category ‘other’. All details regarding frequency of occurrence, average plastic number and mass were published within earlier publications (Van Franeker and Bell 1988; Van Franeker 1985; Van Franeker et al. 2011; Kühn and Van Franeker 2012; Trevail et al. 2015).

### Sample analysis

All plastic items encountered in the seabird stomachs were individually weighed on a Sartorius electronic scale to an accuracy of 0.0001 g. NIR analysis was conducted using a handheld near infrared spectroscope with integrated spectrum library containing 28 different polymer types (for details see Online Supplement Table 2). Some samples from Svalbard were analysed using Agilent Technology 4500a portable FTIR (32 scans, Library: Aarhus University microplastics). Results were accepted based on the match score threshold level identified during the NIR/FTIR experiment. The 10 most occurring polymer types are given in full detail. Other polymer types had a very low occurrence throughout the total sample (< 15 particles, each type representing < 0.3%) and were combined in the category ‘other’.

### Data analysis

For visual impressions, graphs are presented as stacked columns either for numbers or mass. As mass of plastic is considered more important in terms of potential harm (Van Franeker et al. 2011; Provencher et al. 2019) and is used in the framework of monitoring of plastics in fulmars (OSPAR 2015), most graphs depict composition mass percentages. However, all underlying data (numbers and mass) are provided in Online Supplement Table 3.

Tests for statistical significance in polymer type proportions were conducted with https://epitools.ausvet.com.au/ as recommended by Provencher et al. (2017). We compared sample proportions of polymer types with 2-sample *z* test (two-tailed, significance level set at *p* ≤ 0.05; https://epitools.ausvet.io/ztesttwo). This test compares proportional abundance by number of particles and does not consider mass.

## Results

### NIR and FTIR method evaluation

Detailed information on decisions for each test item can be found in Online Supplement Table 1. For synthetic samples, FTIR identified slightly more items correctly than NIR (FTIR: 78, 79, 71 items against NIR: 79, 74, 64 items for 70%, 80% and 90% thresholds respectively; Table 3). In contrast, FTIR failed regularly in distinguishing between plastic and natural items. Out of 83 natural items FTIR misidentified approximately one-half (40 items) even at the highest match score threshold level of 90%. When the threshold was set at 80% or 70%, half of the items were falsely identified as plastics (72 and 73 items, respectively). Falsely identified items include common prey remains such as bones, eye lenses, feathers, skin, crustacean carapaces, squid and polychaete jaws and insect shields. Most of these items were misidentified by FTIR as being polyamide (PA). NIR in general showed lower match scores for natural items, correctly classifying those as not being synthetic polymers. Only at the 70% match score threshold, 15 natural items were misidentified as being plastic, again mainly as PA. Yamashita et al. (2011) described difficulties with measuring dark items, when using NIR which is similar to our findings.

**Table 3:** Number and percentage of items measured with FTIR and NIR with difference match score thresholds (A > 70%, B > 80%, C > 90%)

A					
Match score threshold > 70%		FTIR		NIR	
		*n*	%	*n*	%
Plastic (117)	Correct ID	*78*	66.67	*79*	67.52
	False ID	*32*	27.35	*23*	19.66
	No ID	*7*	5.98	*15*	12.82
Natural (83)	Correct ID	*10*	12.05	*68*	81.93
	False ID	*73*	87.95	*15*	18.07
	No ID	*0*	0.00	*0*	0.00
All (200)	Correct ID	*88*	44.00	*147*	73.50
	False ID	*105*	52.50	*38*	19.00
	No ID	*7*	3.50	*15*	7.50
B					
Match score threshold > 80%		FTIR		NIR	
		*n*	%	*n*	%
Plastic (117)	Correct ID	*79*	67.52	*74*	63.25
	False ID	*27*	23.08	*17*	14.53
	No ID	*11*	9.40	*26*	22.22
Natural (83)	Correct ID	*11*	13.25	*80*	96.39
	False ID	*72*	86.75	*3*	3.61
	No ID	*0*	0.00	*0*	0.00
All (200)	Correct ID	*90*	45.00	*154*	77.00
	False ID	*99*	49.50	*20*	10.00
	No ID	*11*	5.50	*26*	13.00
C					
Match score threshold > 90%		FTIR		NIR	
		*n*	%	*n*	%
Plastic (117)	Correct ID	*71*	60.68	*64*	54.70
	False ID	*11*	9.40	*5*	4.27
	No ID	*35*	29.91	*48*	41.03
Natural (83)	Correct ID	*41*	49.40	*83*	100.00
	False ID	*40*	48.19	*0*	0.00
	No ID	*2*	2.41	*0*	0.00
All (200)	Correct ID	*112*	56.00	*147*	73.50
	False ID	*51*	25.50	*5*	2.50
	No ID	*37*	18.50	*48*	24.00

Each category (synthetic, natural and the combination of both) is divided in items correctly identified, items falsely identified and items not identified

Our results indicate that a high match score threshold for FTIR is necessary to avoid misidentification of natural material (Table 3). At 80% match score threshold, almost half of all items were identified correctly (90/200 test items). For NIR, the match score does not have a significant influence on the reliability of the outcome of synthetic polymers (*p* > 0.4), but a strong influence on the recognition of natural materials. Almost all natural materials (80 out of 83 natural items) were identified as such when a threshold of > 80% match score was applied for NIR which is significantly higher than with a match score of 70% (68 out of 83 natural items; *p* = 0.0027).

Numbers for correct identification vary between the three thresholds. In weighing the potential misidentification of natural material against the successful identification of polymers in the synthetic material category, we have decided to set the threshold at > 80% in the further description of our results.

### Plastic in procellariform seabirds

**Fig. 1:**
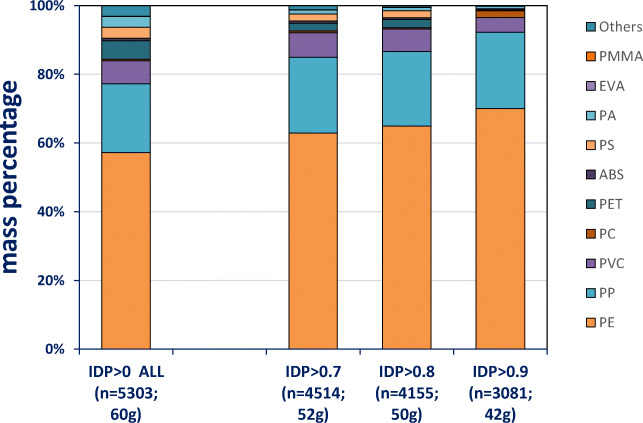
Polymer identification with NIR, applying different match score thresholds of > 70, > 80 and > 90% (shown as identification probabilities (IDP)). Proportion percentages are shown in terms of mass for all plastic items encountered in all seabird samples

A total of 5303 plastic pieces with a combined mass of 60 g was analysed. To evaluate the impact of using the > 80% spectrum match, Fig. 1 shows results when no restriction is applied and when the 70%, 80% or 90% match thresholds are used. Polyethylene and polypropylene (PP) identification became more important when higher thresholds are applied, indicating that spectra for less common polymers show less similarity to those in the polymer library. Using > 80% spectra match threshold, 4155 of plastic pieces and 50 g of plastic mass were identified to a specific polymer type. Thus, 1148 plastic pieces (22% of items) with a combined mass of 10 g (17% of mass) remained below this threshold. Lacking an accepted identification, they are not included in the further analyses of plastic type proportions.

Plastic items belonged to seven categories (Table 4). As already experienced during the testing phase, NIR had difficulties with identifying foams. Of the 739 foam items measured, only 42.5% could be identified. Even though the identification of threads scored high in the testing phase (77% were correctly identified), in more than half of the threads measured in bird stomachs (53.8%), the polymer type remained unclear.

**Table 4:** Categories of marine debris items collected from all birds used in this study

Species	Country	*n* plastics	Fragment	Pellet	Sheet	Thread	Foam	Other	Bead
Northern fulmar	Netherlands	3439	1252	650	657	190	579	111	0
N. fulmar	*NL1980-89*	*(778)*	*(213)*	*(356)*	*(69)*	*(43)*	*(89)*	*(8)*	*(0)*
N. fulmar	*NL1990-99*	*(1129)*	*(389)*	*(94)*	*(378)*	*(83)*	*(97)*	*(88)*	*(0)*
N. fulmar	*NL2010-11*	*(1532)*	*(650)*	*(200)*	*(210)*	*(64)*	*(393)*	*(15)*	*(0)*
N. fulmar	Faroe Islands	536	361	36	49	35	50	4	1
N. fulmar	Iceland	342	199	14	39	25	55	1	9
N. fulmar	Svalbard	595	428	16	47	76	24	3	1
N. fulmar	Total	4912	2240	716	792	326	708	119	11
Wilson’s storm petrel	Antarctica	342	194	110	14	2	20	0	2
Cape petrel	Antarctica	37	15	11	0	1	10	0	0
S. fulmar	Antarctica	11	10	1	0	0	0	0	0
Snow petrel	Antarctica	1	0	0	0	0	1	0	0
Antarctica	Total	391	219	122	14	3	31	0	2
All birds	Total	**5303**	**2459**	**838**	**806**	**329**	**739**	**119**	**13**

**Fig. 2:**
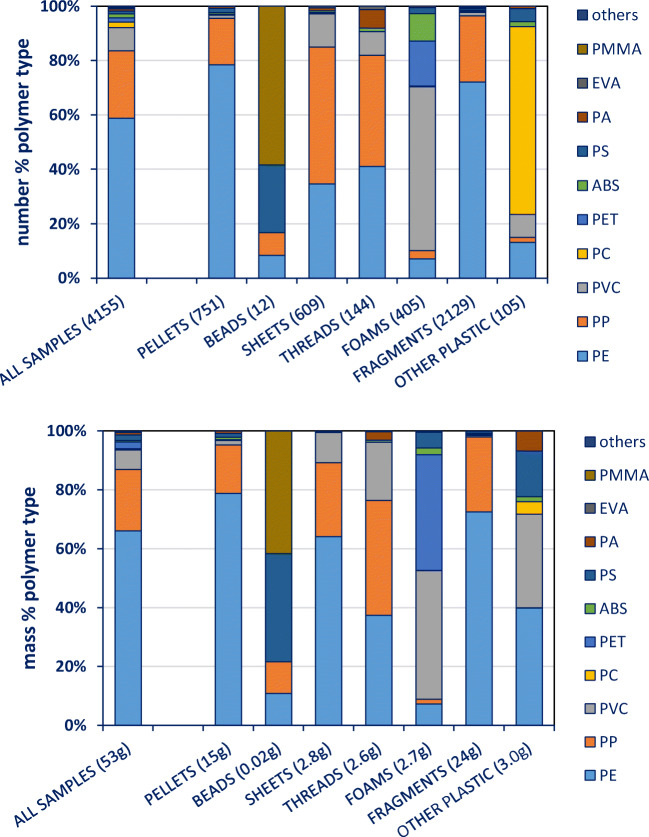
Polymer type proportions of all items identified in this study, belonging to different plastic categories. Top: polymer type proportions in numbers. Bottom: polymer type proportions in gram. Graph is based on data shown in Online Supplement Table 3.2a

We present the number, mass and polymer composition of the plastic particles that were successfully identified and indicate a relationship of plastic category and polymer types (Fig. 2). Data shown in Fig. 2 clearly indicate a relationship of plastic category and polymer types. Pellets and fragments mainly consisted of PE (79 and 72% in terms of mass, respectively), while the other categories showed more variation. Threads, for example, consisted of 37% PE, 39% PP and 20% PVC. In contrast, microbeads were mainly made of PMMA (42%) and PS (37%), but due to their low abundance, they did not strongly influence overall results. Plastics with a high density, such as PVC, influenced the patterns and caused differences between numbers and mass (e.g. PVC in the ‘other plastic’ category (Fig. 2)).

As the variation between plastic categories was substantial, spatial and temporal, comparisons are presented separately per plastic category as well.

### Temporal comparison

**Fig. 3:**
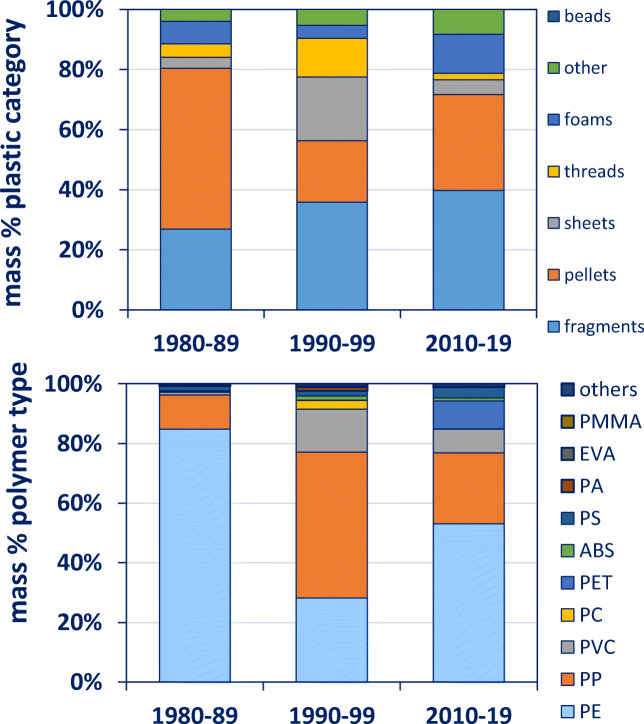
Top: plastic categories ingested by northern fulmars in three decennia. Bottom: polymer categories of these plastics. Both graphs show proportions in terms of mass. Graph is based on data shown in Online Supplement Table 3.3a and b

Plastics in fulmars from the Netherlands have been studied from the 1980s onwards. Here we compare plastics ingested by northern fulmars from three decennia. Pellets comprised the majority of mass (54%) during the 1980s (Fig. 3). In the 1990s, the mass of fragments, sheet and threads increased, and consequently the proportion of pellets decreased (20%). However, in the 2010s, pellets again gained relative importance (32%) together with fragments (40%) and foam (13%), possibly influenced by a fulmar from 2010 with an unusual number of ingested pellets (*n* = 72). In the 1980s, 85% of the plastics were made of PE and 11% of PP, and only 4% were made of other polymer types (Fig. 3). In the 1990s, more diversity in plastic types was observed (14.4% of PVC and 2.9% polycarbonate (PC)). Polypropylene comprised almost half of the plastics (49%). In the most recent decennia, PE gained relative importance (53%), while PC almost disappeared (0.3%). Polyethylene terephthalate (PET) and polystyrene (PS) increased (9.4 and 7.9%, respectively). Numbers of PE differed significantly for all three decennia (p < 0.0003).

### Regional comparison in the North Atlantic

**Fig. 4:**
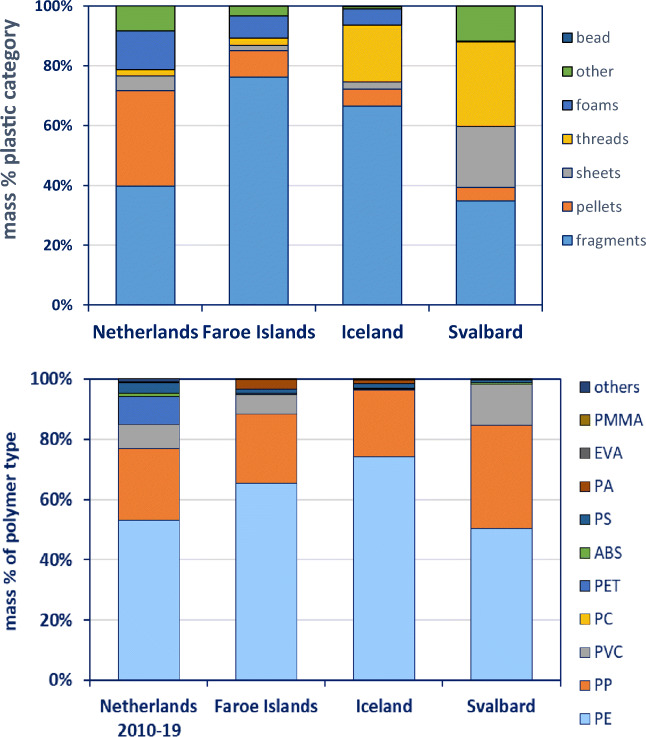
Top: plastic categories ingested by northern fulmars from four North Atlantic regions. Bottom: polymer categories of these plastics. Both graphs show proportions in terms of mass. Graph is based on data shown in Online Supplement Table 3.4a and b

Data for northern fulmars is available for four regions in the North Atlantic: the Netherlands, the Faroe Islands, Iceland and Svalbard (Table 4; Fig. 4). For the Netherlands, only data from the most recent decennia (2010–2019) was included, as data from the other regions were collected between 2011 and 2013. Fragments were the most dominant plastic category in all regions; for the Faroe Islands and Iceland, more than half of the plastic mass consisted of fragments (Fig. 4). Pellets were more common in the Netherlands but decreased with higher latitude. Threads were comparably abundant in Iceland and Svalbard. The highest variation of plastic types was found in the Netherlands. Polyethylene and PP comprised the majority of plastic mass in all regions and increased with latitude, with the exception of Svalbard, where the proportions of PE mass seemed closer to those of the Netherlands (Fig. 4). However, when testing for significant differences in PE numbers, the Netherlands had significantly less PE plastic items than all other locations (*p* < 0.0001). The Faroe Islands, Iceland and Svalbard did not show significant differences in the number of PE items (*p* > 0.1). Noticeably PVC comprised 13% of mass on Svalbard but much lower proportions in the Netherlands, the Faroe Islands and Iceland (8%, 6% and 0%, respectively).

### Global comparison

**Fig. 5:**
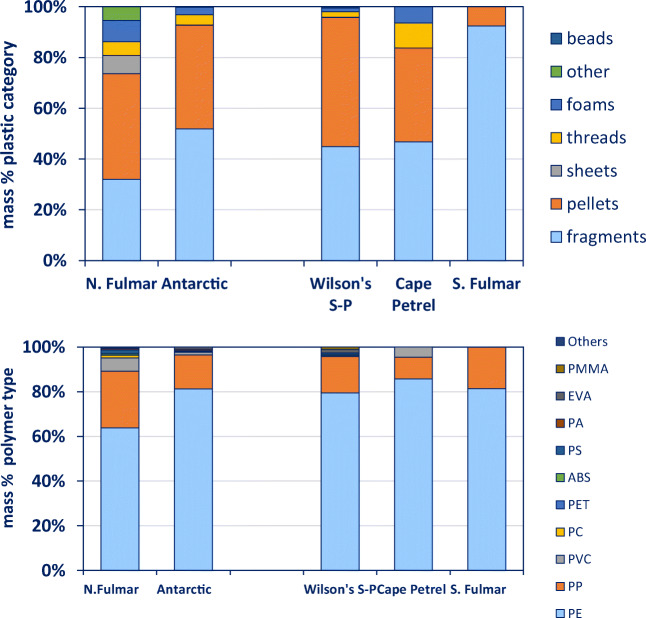
Top: plastic categories ingested by northern fulmars from the 1980s to the 1990s and related seabird species from the Southern Ocean (all southern species combined and separately per species). Bottom: polymer categories of these plastics. Both graphs show proportions in terms of mass. Graph is based on data shown in Online Supplement Table 3.3a (N. fulmars) and 3.5a and b.

Sample numbers for the southern species were, except for the Wilsons’s storm petrel, very low. There was one snow petrel available that had ingested one item (which could not be identified at > 80% match score). For northern fulmars, only data from the 1980s and 1990s were used to ensure comparability. Variation in plastic categories in northern fulmars was higher with sheets, thread and other plastics, while the Antarctic samples were dominated by fragments and pellets only. Polymer compositions (in mass) for the southern species separately and as a group are shown (Fig. 5). In terms of numbers, the polymer composition of the three remaining southern species did not differ significantly (*p* > 0.73) from each other, and therefore, these species were treated as one group. In southern species, PE mass is higher (81%) than in northern fulmars (64%) (Fig. 5). Also, the number of PE items was significantly higher in Antarctic birds than in northern fulmars (*p* < 0.0001).

## Discussion

### Infrared spectroscopy thresholds

FTIR analysis is a method increasingly used to identify plastics ingested by marine organisms. Currently, there is no common agreement on match score threshold levels in IR analysis. Literature research revealed 86 studies that have used either FTIR or NIR to determine the composition of plastics in seabirds, marine mammals, turtles and marine fish (Online Supplement Table 4). The threshold varied between 60 and 93%. Most studies that give details have varied in acceptance rate between 70 and 85% (Alomar and Deudero 2017; Bessa et al. 2018; Ory et al. 2017; Ory et al. 2018; Tanaka and Takada 2016). Unfortunately, a majority of studies using FTIR (*n* = 49; Online Supplement Table 4) did not provide any details on match score thresholds at all. Therefore, it is unclear whether they used a threshold or rather accepted any result displayed. For this study, a threshold level was established for the reliable identification of plastic items ingested by marine organisms, using either FTIR or NIR.

By testing both methods on a series of various natural and plastic items, we decided that in our case, a match score of 80% should be applied to document polymer composition of our samples. It is recommended that validity of match scores should be examined in each study.

The 80% represents a slightly higher threshold level than proposed by Lusher et al. (2017). These authors recommended the (often arbitrarily chosen) average threshold levels used in previous studies (70–75%) as a standard for plastics in fish and invertebrates that usually range in the size of micrometres.

Many different natural items such as fish and squid lenses, beaks, bones, skin and feathers were falsely identified by FTIR as being polymers. Precaution should be taken to avoid overestimation of plastics ingested by organisms, when solely relying on FTIR outcomes. Good background knowledge of typical natural food remains, occurring in the species studied, is highly recommended to avoid misidentification by FTIR. Identification of plastics is further complicated by the nature of plastics originating from biota samples. FTIR penetrates the surface of plastics for only a few micrometres (Renner et al. 2017); therefore, surface degradation and biofouling can cause high background noise in measurements, potentially contributing to the confusion of PA and natural keratin. Although NIR has shown to have difficulties with foams and dark colours, still both FTIR and NIR seem to be reliable and can be recommended for the identification of polymers.

### Polymer types in seabirds

Infrared polymer analysis has not been applied to any of the species researched in this study. Although plastic is common in these species, the only crude polymer identification in Wilson’s storm petrels and northern fulmars has been conducted by Moser and Lee (1992), using density separation. In both species the great majority of ingested plastic items were floating and, according to the authors, belonged to either PE or PP. This overlaps with the results from our findings, where PE and PP were the most common polymer types in both time and space.

Our data indicate that plastic categories determine polymer characteristics more than time and space scale. Fragments are predominantly made of PE (71%) and PP (27%). Characteristics of plastics ingested by birds, such as size and shape, have changed through time (Ryan 2008; Van Franeker and Law 2015). When fragment mass is high in birds, PE and PP are the most abundant polymer type as well. Many pellets in a sample increase the relative PE mass, e.g. in early data from the Netherlands (46%) and in Antarctic seabirds (31%). Since the 1990s, pellets decreased in northern fulmars, while mainly fragment mass increased (Van Franeker and Law 2015) and also PE mass decreased in the same time. Unfortunately no recent data of plastic ingestion by Antarctic seabirds is available that could confirm similar trends in the Southern Ocean. However, two recent studies from the Southern Ocean, investigating microplastics at the water surface, found PE and PP to be the dominant polymer types in non-fibrous plastics (Cincinelli et al. 2017; Isobe et al. 2017).

**Fig. 6:**
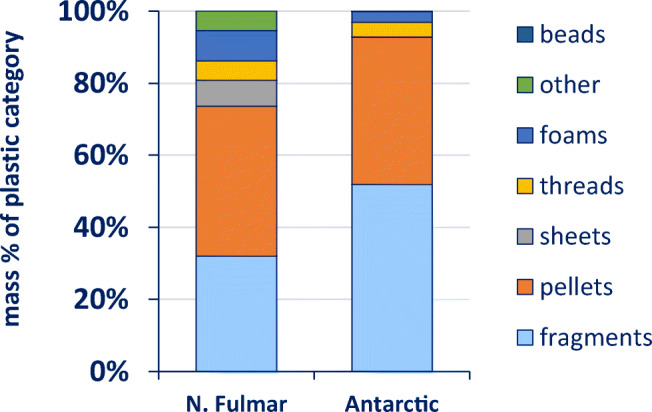
Number of plastics in northern fulmars from the 1980s to the 1990s and procellariform seabirds from Antarctica per plastic category. Graph is based on data shown in Online Supplement Table 3.3a (N. fulmars) and 3.5a

Threads were mainly found in Iceland and Svalbard, both remote places, where fishery-related plastic dominates the litter found on beaches (Bergmann et al. 2017; Falk-Andersson and Strietman 2019). The majority of threads (in terms of mass) ingested by all northern fulmars in the North Atlantic were made of PE (49%) and PP (21%). Only 3% of ingested threads comprised of PA (Online Supplement Table 3.3b), probably explained by the high density of PA, causing net and rope material made of PA to sink out of reach of surface-foraging seabirds. Soft materials such as sheets and foam are less abundant in birds from the Southern Ocean (Fig. 6). Seabirds migrate from their more polluted wintering areas to their breeding colonies in Antarctica (Van Franeker and Law 2015). Soft material digests quicker than hard pellets or fragments (Ryan 2015), and sheets and foam may therefore be excreted when arriving on their breeding locations on the continent. Soft plastic items may also disappear from the ocean’s surface and therefore out of the reach of fulmarine petrels, before reaching the Southern Ocean (Suaria et al. 2020).

Foam in northern fulmar samples was identified as mainly being PVC (59% in terms of number, 42% of mass). PS comprised of only 5% in numbers and 2% of mass of foams found in the fulmar stomachs. A possible explanation might be the extremely low specific mass of PS foams, which may lead to rapid disappearance from open ocean environments where fulmars forage. Different rates of processing on the materials in the bird’s stomachs are another speculative interpretation. PVC was the predominant polymer type ingested by little auks (*Alle alle*) from the Arctic (Amélineau et al. 2016); however, almost all plastics (97.2%) the authors found were microfibres, a plastic category not considered in our study. PVC is a plastic type, usually containing high quantities of phthalate plasticizers (Hermabessiere et al. 2017). These substances are known for leaching from the plastics and for their endocrine disruptive characteristics (Oehlmann et al. 2009). PVC is the third most common plastic type in our seabird sample, and the associated risks of ingesting PVC should be of concern (Rochman 2015).

In seabirds, no polymer type specific preference has been reported. Plastic uptake by seabirds might simply reflect the background availability of plastics produced through the time and the distribution of marine plastic debris on the ocean surface. Polyethylene and PP are less dense than seawater, and almost all polymers, including those heavier than seawater, can be found in their expanded form as foam. This might explain the great availability of these polymers for the uptake by surface seizing seabirds. From the beginning of the industrial plastic production in the 1950s and onwards, PE and PP have always been the most commonly produced polymers over time (Geyer et al. 2017), resulting in widespread disposal.

## Conclusion

FTIR has become a common identification method in plastic research. NIR has been used to a lesser extent; however, both methods are suitable for plastic ingestion studies. Caution should be given to the fact that especially FTIR tends to misidentify natural hard prey items as being plastic, mostly as synthetic polyamide. In order to reduce this type of error, we decided to use a reliability threshold of 80% with library matches. Basic knowledge of natural diet of the organism studied is valuable in order to evaluate the risk of small food fragments being misidentified as a synthetic polymer. Our results of plastic ingested by seabirds indicate a general predominance of PE and PP polymers, but the plastic category available at a specific location or in a specific time frame seems to be the driving factor of polymer proportions in seabirds, rather than variations in preference for different plastic types.

## Electronic supplementary material

ESM 1 (DOCX 294 kb)
