# Supplementary material for: Polymer types ingested by northern fulmars (Fulmarus glacialis) and southern hemisphere relatives
Source: Environ Sci Pollut Res Int. 2020 Aug 26;28(2):1643–55. doi: 10.1007/s11356-020-10540-6 (PMC7785538; doi:10.1007/s11356-020-10540-6)
Supplement: Supplementary file 1 — (DOCX 294 kb) [file 11356_2020_10540_MOESM1_ESM.docx]

*Online Supplement to:*

# Global and temporal variation of polymer types ingested by the northern fulmar and southern hemisphere relatives

Susanne Kühn^1*^, Albert van Oyen^2^, Elisa L. Bravo Rebolledo^1,3^, Amalie V. Ask^4^, Jan Andries van Franeker^1^

^1^ Wageningen Marine Research, Ankerpark 27, 1781 AG Den Helder, The Netherlands

^2^ CARAT GmbH, Harderhook 22, 46395 Bocholt, Germany

^3^ Bureau Waardenburg bv, Varkensmarkt 9, 4101 CK Culemborg, The Netherlands

^4^ Norwegian Polar Institute, Fram Centre, Post box 6606 Langnes, N-9296 Tromsø, Norway

**Contact:** [susanne.kuehn@wur.nl](mailto:susanne.kuehn@wur.nl)

Contents

[Global and temporal variation of polymer types ingested by the northern fulmar and southern hemisphere relatives 1](#_Toc42853913)

[Online Supplement Table 1: Details NIR and FTIR comparison 2](#_Toc42853914)

[Online Supplement Table 2 Polymer type abbreviations 16](#_Toc42853915)

[Online Supplement Table 3: Details on plastic categories and polymer types found in this study 17](#_Toc42853916)

[Online Supplement Table 4. Details on polymer identification methods using infrared spectroscopy and match score thresholds applied in different studies. 23](#_Toc42853917)

[Online Supplement References 23](#_Toc42853918)

## Online Supplement Table 1: Details NIR and FTIR comparison

Plastics with polymer type and natural items analysed with FTIR and NIR. Details on plastic category and colour are given. For FTIR and NIR results are presented as ID match (plastic type with highest match score), the match score (0-100) with three different match score thresholds (>90%, >80% and >70%). All items were categorized as correct ID (correct polymer/natural type and match score higher than intended match score; No ID (lower match score than intended) and false ID (false polymer type but match score higher than intended match score).

| Sample number | Polymer type | Object description | Category | Colour | Type | synthetic (1), natural (0) | FTIR ID | FTIR match score | Correct ID >90% | Correct ID >80% | Correct ID >70% | No ID >90% | No ID >80% | No ID >70% | False ID >90% | False ID >80% | False ID >70% | NIR ID | NIR match score | Correct ID >90% | Correct ID >80% | Correct ID >70% | No ID >90% | No ID >80% | No ID >70% | False ID >90% | False ID >80% | False ID >70% |
| --- | --- | --- | --- | --- | --- | --- | --- | --- | --- | --- | --- | --- | --- | --- | --- | --- | --- | --- | --- | --- | --- | --- | --- | --- | --- | --- | --- | --- |
| CRT-TEST-001 | PET | Water Bottle | fragment | light blue transparent | consumer plastic | 1 | PET | 98.3 | 1 | 1 | 1 |  |  |  |  |  |  | PET | 97 | 1 | 1 | 1 |  |  |  |  |  |  |
| CRT-TEST-002 |  | Chemical (Texel, July 2013; paraffine like) | other | white | paraffine | 1 | LDPE | 95.5 |  |  |  |  |  |  | 1 | 1 | 1 | PE | 96 |  |  |  |  |  |  | 1 | 1 | 1 |
| CRT-TEST-003 | PVC | Waterpipe | fragment | grey | consumer plastic | 1 | PVC | 95.2 | 1 | 1 | 1 |  |  |  |  |  |  | PP | 72 |  |  |  | 1 | 1 |  |  |  | 1 |
| CRT-TEST-004 |  | Chemical (Texel, July 2013; hard) | other | white | paraffine | 1 | Ionomer 2 | 84.7 | 1 |  |  |  |  |  |  | 1 | 1 | PE | 74 | 1 | 1 |  |  |  |  |  |  | 1 |
| CRT-TEST-005 |  | Fulmar foot web (skin) |  |  |  | 0 | PA6/66 GF FR | 92.1 |  |  |  |  |  |  | 1 | 1 | 1 | PA | 67 | 1 | 1 | 1 |  |  |  |  |  |  |
| CRT-TEST-006 | PS | Styrofoam | foam | whitish | consumer plastic | 1 | HIPS | 97.7 | 1 | 1 | 1 |  |  |  |  |  |  | ABS | 89 |  |  |  | 1 |  |  |  | 1 | 1 |
| CRT-TEST-007 | SAN | School cup | fragment | colourprinted white | consumer plastic | 1 | PVC | 77.6 |  |  |  | 1 | 1 |  |  |  | 1 | ABS | 90 |  |  |  |  |  |  | 1 | 1 | 1 |
| CRT-TEST-008 |  | plant - hard stem (reed like) |  |  |  | 0 | Arabic | 86.9 | 1 |  |  |  |  |  |  | 1 | 1 | PA | 51 | 1 | 1 | 1 |  |  |  |  |  |  |
| CRT-TEST-009 | PET | Food container | fragment | green | consumer plastic | 1 | LDPE | 95.8 |  |  |  |  |  |  | 1 | 1 | 1 | PET | 88 |  | 1 | 1 | 1 |  |  |  |  |  |
| CRT-TEST-010 | PE | Bag electric equipment | sheet | black printed transparent | consumer plastic | 1 | LDPE | 93.9 | 1 | 1 | 1 |  |  |  |  |  |  | PE | 92 | 1 | 1 | 1 |  |  |  |  |  |  |
| CRT-TEST-011 | PVC | vGansewinkel reference material | fragment | coloured | raw material | 1 | ABS | 86.3 |  |  |  | 1 |  |  |  | 1 | 1 | PVC | 78 |  |  | 1 | 1 | 1 |  |  |  |  |
| CRT-TEST-012 | PE | Bag | sheet | pink | consumer plastic | 1 | LDPE | 94.8 | 1 | 1 | 1 |  |  |  |  |  |  | PE | 93 | 1 | 1 | 1 |  |  |  |  |  |  |
| CRT-TEST-013 |  | Shellfish - Blue Mussel |  |  |  | 0 | PA HTN | 91.5 |  |  |  |  |  |  | 1 | 1 | 1 | PA | 53 | 1 | 1 | 1 |  |  |  |  |  |  |
| CRT-TEST-014 | PS | Meat tray | fragment | transparent | consumer plastic | 1 | PS | 97.3 | 1 | 1 | 1 |  |  |  |  |  |  | PS | 93 | 1 | 1 | 1 |  |  |  |  |  |  |
| CRT-TEST-015 | PLA | Compostable food container | fragment | transparent | bio-plastic | 1 | No result |  | 1 | 1 | 1 |  |  |  |  |  |  | PMMA | 65 | 1 | 1 | 1 |  |  |  |  |  |  |
| CRT-TEST-016 |  | Fish goby (small dried) |  |  |  | 0 | PA6/66 GF FR | 89.5 | 1 |  |  |  |  |  |  | 1 | 1 | PACC | 58 | 1 | 1 | 1 |  |  |  |  |  |  |
| CRT-TEST-017 | PET | Food container | fragment | dirty transparent | consumer plastic | 1 | PET | 98 | 1 | 1 | 1 |  |  |  |  |  |  | PET | 97 | 1 | 1 | 1 |  |  |  |  |  |  |
| CRT-TEST-018 |  | Sprat fish bone |  |  |  | 0 | Arabic | 88 | 1 |  |  |  |  |  |  | 1 | 1 | PA | 43 | 1 | 1 | 1 |  |  |  |  |  |  |
| CRT-TEST-019 | PVC | vGansewinkel reference material | sheet | transparent | raw material | 1 | PVC | 87.5 |  | 1 | 1 | 1 |  |  |  |  |  | PVC | 93 | 1 | 1 | 1 |  |  |  |  |  |  |
| CRT-TEST-020 |  | Tomato skin |  |  |  | 0 | Arabic | 88.3 | 1 |  |  |  |  |  |  | 1 | 1 | PA | 88 | 1 |  |  |  |  |  |  | 1 | 1 |
| CRT-TEST-021 | PP | CD box bottom | fragment | grey | consumer plastic | 1 | PP Homo | 96.9 | 1 | 1 | 1 |  |  |  |  |  |  | PP | 96 | 1 | 1 | 1 |  |  |  |  |  |  |
| CRT-TEST-022 |  | Fulmar feather 2 |  |  |  | 0 | PA HTN | 89.7 | 1 |  |  |  |  |  |  | 1 | 1 | PA/ABS | 42 | 1 | 1 | 1 |  |  |  |  |  |  |
| CRT-TEST-023 | 7P0342 | Compostable potato bag | sheet | lightly coloured | bio-plastic | 1 | PBT/PET GF | 86.4 | 1 |  |  |  |  |  |  | 1 | 1 | PVC | 42 | 1 | 1 | 1 |  |  |  |  |  |  |
| CRT-TEST-024 |  | Angler bone fragment |  |  |  | 0 | PA HTN | 93 |  |  |  |  |  |  | 1 | 1 | 1 | PA | 53 | 1 | 1 | 1 |  |  |  |  |  |  |
| CRT-TEST-025 | PC | Sample jar | fragment | transparent | consumer plastic | 1 | PC | 99.6 | 1 | 1 | 1 |  |  |  |  |  |  | PC | 97 | 1 | 1 | 1 |  |  |  |  |  |  |
| CRT-TEST-026 | Silica | Silica gel desiccant | pellet | transparent | consumer plastic | 1 | Silicon | 88 |  | 1 | 1 | 1 |  |  |  |  |  | PETG | 37 | 1 | 1 | 1 |  |  |  |  |  |  |
| CRT-TEST-027 | Cradonyl | Compostable pellet | pellet | white | bio-plastic | 1 | PA | 80.2 | 1 |  |  |  |  |  |  | 1 | 1 | CA | 84 |  |  |  | 1 |  |  |  | 1 | 1 |
| CRT-TEST-028 | Century | Bioldegradable spoon | fragment | whitish | bio-plastic | 1 | Arabic | 82.6 |  | 1 | 1 | 1 |  |  |  |  |  | PA | 83 |  |  |  | 1 |  |  |  | 1 | 1 |
| CRT-TEST-029 |  | Fish eyelens whiting |  |  |  | 0 | PA HTN | 93.4 |  |  |  |  |  |  | 1 | 1 | 1 | PA | 70 | 1 | 1 |  |  |  |  |  |  | 1 |
| CRT-TEST-030 | PE | vGansewinkel reference material | fragment | blue | raw material | 1 | LDPE | 93.7 | 1 | 1 | 1 |  |  |  |  |  |  | PE | 95 | 1 | 1 | 1 |  |  |  |  |  |  |
| CRT-TEST-031 |  | Angler oogbol |  |  |  | 0 | PA HTN | 86.4 | 1 |  |  |  |  |  |  | 1 | 1 | PA | 23 | 1 | 1 | 1 |  |  |  |  |  |  |
| CRT-TEST-032 | PVC | Waterpipe | fragment | grey | consumer plastic | 1 | PVC | 95.3 | 1 | 1 | 1 |  |  |  |  |  |  | PP | 64 | 1 | 1 | 1 |  |  |  |  |  |  |
| CRT-TEST-033 |  | Crab, shield fragment |  |  |  | 0 | PA HTN | 84.1 | 1 |  |  |  |  |  |  | 1 | 1 | PA | 41 | 1 | 1 | 1 |  |  |  |  |  |  |
| CRT-TEST-034 | PE | vGansewinkel reference material | fragment | red | raw material | 1 | LDPE | 91.9 | 1 | 1 | 1 |  |  |  |  |  |  | PE | 94 | 1 | 1 | 1 |  |  |  |  |  |  |
| CRT-TEST-035 |  | Fulmar toe nail |  |  |  | 0 | PA66 | 89 | 1 |  |  |  |  |  |  | 1 | 1 | PA | 38 | 1 | 1 | 1 |  |  |  |  |  |  |
| CRT-TEST-036 | PE | vGansewinkel reference material | fragment | black | raw material | 1 | EMA | 93 | 1 | 1 | 1 |  |  |  |  |  |  | PS | 13 |  |  |  | 1 | 1 | 1 |  |  |  |
| CRT-TEST-037 | PP | Wrapper cellophane-like Post-it notes | sheet | orange printed transparent | consumer plastic | 1 | PMMA | 88.2 | 1 |  |  |  |  |  |  | 1 | 1 | PP | 91 | 1 | 1 | 1 |  |  |  |  |  |  |
| CRT-TEST-038 | Cradonyl | compostable fragment | fragment | redbrown | bio-plastic | 1 | Polyarylate | 77.9 | 1 | 1 |  |  |  |  |  |  | 1 | PMMA | 81 |  |  |  | 1 |  |  |  | 1 | 1 |
| CRT-TEST-039 | PP | Wrapper dishwash tablet | sheet | printed transparent | consumer plastic | 1 | PA6 | 85.8 | 1 |  |  |  |  |  |  | 1 | 1 | PP | 89 |  | 1 | 1 | 1 |  |  |  |  |  |
| CRT-TEST-040 | Cradonyl | ompostable fragment | fragment | whitish | bio-plastic | 1 | Arabic | 80.3 |  | 1 | 1 | 1 |  |  |  |  |  | PMMA | 81 |  |  |  | 1 |  |  |  | 1 | 1 |
| CRT-TEST-041 |  | Harbour Seal hair |  |  |  | 0 | PA HTN | 91.9 |  |  |  |  |  |  | 1 | 1 | 1 | PA | 60 | 1 | 1 | 1 |  |  |  |  |  |  |
| CRT-TEST-042 | PET | vGansewinkel reference material | fragment | transparent | raw material | 1 | PET | 97.4 | 1 | 1 | 1 |  |  |  |  |  |  | PET | 97 | 1 | 1 | 1 |  |  |  |  |  |  |
| CRT-TEST-043 | PE | vGansewinkel reference material | sheet | transparent | raw material | 1 | LDPE | 95.1 | 1 | 1 | 1 |  |  |  |  |  |  | PE | 92 | 1 | 1 | 1 |  |  |  |  |  |  |
| CRT-TEST-044 | PA | SUIT fishing net | thread | dark green | consumer plastic | 1 | PA6 GF | 94.6 | 1 | 1 | 1 |  |  |  |  |  |  | PA | 81 |  | 1 | 1 | 1 |  |  |  |  |  |
| CRT-TEST-045 | PMMA | vGansewinkel reference material | fragment | white | raw material | 1 | PMMA | 99.5 | 1 | 1 | 1 |  |  |  |  |  |  | PMMA | 92 | 1 | 1 | 1 |  |  |  |  |  |  |
| CRT-TEST-046 | PC | vGansewinkel reference material | fragment | transparent | raw material | 1 | PC | 99.7 | 1 | 1 | 1 |  |  |  |  |  |  | PC | 95 | 1 | 1 | 1 |  |  |  |  |  |  |
| CRT-TEST-047 | PS | Food container | fragment | transparent | consumer plastic | 1 | HIPS | 98.8 | 1 | 1 | 1 |  |  |  |  |  |  | PS | 95 | 1 | 1 | 1 |  |  |  |  |  |  |
| CRT-TEST-048 |  | Shellfish - Oyster |  |  |  | 0 | Melamin | 80.7 | 1 |  |  |  |  |  |  | 1 | 1 | PA | 11 | 1 | 1 | 1 |  |  |  |  |  |  |
| CRT-TEST-049 | PP | vGansewinkel reference material | fragment | thick semitransparent sheet |  | 1 | PP Homo | 98.2 | 1 | 1 | 1 |  |  |  |  |  |  | PP | 99 | 1 | 1 | 1 |  |  |  |  |  |  |
| CRT-TEST-050 |  | Fulmar feather 1 |  |  |  | 0 | PA HTN | 94.1 |  |  |  |  |  |  | 1 | 1 | 1 | PA | 55 | 1 | 1 | 1 |  |  |  |  |  |  |
| CRT-TEST-051 | PS | Food container | fragment | transparent | consumer plastic | 1 | PS | 98 | 1 | 1 | 1 |  |  |  |  |  |  | PS | 95 | 1 | 1 | 1 |  |  |  |  |  |  |
| CRT-TEST-052 |  | Whelk eggs |  |  |  | 0 | Arabic | 90.1 |  |  |  |  |  |  | 1 | 1 | 1 | PA | 48 | 1 | 1 | 1 |  |  |  |  |  |  |
| CRT-TEST-053 | PVC | vGansewinkel reference material | fragment | Blue | raw material | 1 | ABS | 87.7 |  |  |  | 1 |  |  |  | 1 | 1 | PVC | 76 |  |  | 1 | 1 | 1 |  |  |  |  |
| CRT-TEST-054 |  | Crab, leg fragment |  |  |  | 0 | Aramid | 87.3 | 1 |  |  |  |  |  |  | 1 | 1 | PA | 41 | 1 | 1 | 1 |  |  |  |  |  |  |
| CRT-TEST-055 | PS | vGansewinkel reference material | fragment | brown | raw material | 1 | PS | 98.5 | 1 | 1 | 1 |  |  |  |  |  |  | PS | 95 | 1 | 1 | 1 |  |  |  |  |  |  |
| CRT-TEST-056 |  | Fulmar gizzard lining |  |  |  | 0 | PA HTN | 90.8 |  |  |  |  |  |  | 1 | 1 | 1 | PA | 71 | 1 | 1 |  |  |  |  |  |  | 1 |
| CRT-TEST-057 | PET | PET - Sourcy Water Bottle | fragment | very light blue transparent | consumer plastic | 1 | PET | 98.1 | 1 | 1 | 1 |  |  |  |  |  |  | PET | 96 | 1 | 1 | 1 |  |  |  |  |  |  |
| CRT-TEST-058 | PP | PP - wrapper cellofaan like Post-it notes | sheet | transparent | consumer plastic | 1 | PMMA | 89.4 |  |  |  | 1 |  |  |  | 1 | 1 | PP | 72 |  |  | 1 | 1 | 1 |  |  |  |  |
| CRT-TEST-059 |  | Sheepwool natural from dyke |  |  |  | 0 | PA HTN | 93.1 |  |  |  |  |  |  | 1 | 1 | 1 | PA | 56 | 1 | 1 | 1 |  |  |  |  |  |  |
| CRT-TEST-060 | SAN | School cup | fragment | white | consumer plastic | 1 | SAN | 94.6 | 1 | 1 | 1 |  |  |  |  |  |  | ABS | 91 |  |  |  |  |  |  | 1 | 1 | 1 |
| CRT-TEST-061 |  | wood - branch part |  |  |  | 0 | Arabic | 90.7 |  |  |  |  |  |  | 1 | 1 | 1 | PS | 12 | 1 | 1 | 1 |  |  |  |  |  |  |
| CRT-TEST-062 | PE | wrapper paper tissues | sheet | red | consumer plastic | 1 | LDPE | 95.9 | 1 | 1 | 1 |  |  |  |  |  |  | PE | 92 | 1 | 1 | 1 |  |  |  |  |  |  |
| CRT-TEST-063 |  | Fulmar bill 1 |  |  |  | 0 | PA HTN | 92.2 |  |  |  |  |  |  | 1 | 1 | 1 | PA | 72 | 1 | 1 |  |  |  |  |  |  | 1 |
| CRT-TEST-064 | PS | Coffee cup | fragment | white | consumer plastic | 1 | GPPS | 97.3 | 1 | 1 | 1 |  |  |  |  |  |  | PS | 95 | 1 | 1 | 1 |  |  |  |  |  |  |
| CRT-TEST-065 |  | Shellfish - Cockle |  |  |  | 0 | No result |  | 1 | 1 | 1 |  |  |  |  |  |  | POM | 5 | 1 | 1 | 1 |  |  |  |  |  |  |
| CRT-TEST-066 | PP | Water can | fragment | green printed transparent | consumer plastic | 1 | PP Homo | 99 | 1 | 1 | 1 |  |  |  |  |  |  | PP | 98 | 1 | 1 | 1 |  |  |  |  |  |  |
| CRT-TEST-067 |  | Seaweed (Ulva) |  |  |  | 0 | Arabic | 88.5 | 1 |  |  |  |  |  |  | 1 | 1 | PA | 48 | 1 | 1 | 1 |  |  |  |  |  |  |
| CRT-TEST-068 | PVC | Dipped on cotton (offshore glove) | other | rubbery; blue | consumer plastic | 1 | PVC | 86.9 |  | 1 | 1 | 1 |  |  |  |  |  | PVC | 95 | 1 | 1 | 1 |  |  |  |  |  |  |
| CRT-TEST-069 |  | Cotton - inner part of SHOVA glove | thread | white |  | 0 | Arabic | 87.3 | 1 |  |  |  |  |  |  | 1 | 1 | PA | 25 | 1 | 1 | 1 |  |  |  |  |  |  |
| CRT-TEST-070 |  | Shelfish - Ensis old |  |  |  | 0 | No result |  | 1 | 1 | 1 |  |  |  |  |  |  | PA | 32 | 1 | 1 | 1 |  |  |  |  |  |  |
| CRT-TEST-071 | Polyester | Blue woven band | thread | dark blue | consumer plastic | 1 | PET GF | 98.3 |  |  |  |  |  |  | 1 | 1 | 1 | PET | 79 |  |  |  | 1 | 1 |  |  |  | 1 |
| CRT-TEST-072 |  | jellyfish (dried) |  |  |  | 0 | Skin | 77.5 |  | 1 | 1 | 1 |  |  |  |  |  | PA | 31 | 1 | 1 | 1 |  |  |  |  |  |  |
| CRT-TEST-073 | PA | Tie-wrap | fragment | milky | consumer plastic | 1 | PA66 | 98.6 | 1 | 1 | 1 |  |  |  |  |  |  | PA | 94 | 1 | 1 | 1 |  |  |  |  |  |  |
| CRT-TEST-074 |  | Shellfish (slipper shell) |  |  |  | 0 | Skin | 90.3 |  | 1 | 1 | 1 |  |  |  |  |  | PA | 33 | 1 | 1 | 1 |  |  |  |  |  |  |
| CRT-TEST-075 | Acryl | Acryl Sun sail | thread | Red | consumer plastic | 1 | No result |  |  |  |  | 1 | 1 | 1 |  |  |  | POM | 41 |  |  |  | 1 | 1 | 1 |  |  |  |
| CRT-TEST-076 |  | Angler cartilage |  |  |  | 0 | Arabic | 87 | 1 |  |  |  |  |  |  | 1 | 1 | PA | 57 | 1 | 1 | 1 |  |  |  |  |  |  |
| CRT-TEST-077 | PA | Fishing line (knotted) | thread | yellow | consumer plastic | 1 | PP Block | 96.2 |  |  |  |  |  |  | 1 | 1 | 1 | PP | 87 |  |  |  | 1 |  |  |  | 1 | 1 |
| CRT-TEST-078 |  | Shrimp (dried carapax head) |  |  |  | 0 | Aramid | 86.7 | 1 |  |  |  |  |  |  | 1 | 1 | PA | 65 | 1 | 1 | 1 |  |  |  |  |  |  |
| CRT-TEST-079 |  | Angler tooth |  |  |  | 0 | Arabic | 81.6 | 1 |  |  |  |  |  |  | 1 | 1 | PA | 52 | 1 | 1 | 1 |  |  |  |  |  |  |
| CRT-TEST-080 | PVC | Outdoor chair cover | rubber | whitish | consumer plastic | 1 | PET GF | 80.4 |  |  |  | 1 |  |  |  | 1 | 1 | PVC | 85 |  | 1 | 1 | 1 |  |  |  |  |  |
| CRT-TEST-081 |  | Shellfish - Ensis young |  |  |  | 0 | No result |  | 1 | 1 | 1 |  |  |  |  |  |  | PA | 17 | 1 | 1 | 1 |  |  |  |  |  |  |
| CRT-TEST-082 | PVC | Isolation tape | sheet | blue | consumer plastic | 1 | PVC | 82.5 |  | 1 | 1 | 1 |  |  |  |  |  | PVC | 92 | 1 | 1 | 1 |  |  |  |  |  |  |
| CRT-TEST-083 |  | wood - old |  |  |  | 0 | Arabic | 97.2 |  |  |  |  |  |  | 1 | 1 | 1 | PA | 37 | 1 | 1 | 1 |  |  |  |  |  |  |
| CRT-TEST-084 | PP | Sisal rope imitation | thread | brown | consumer plastic | 1 | PP | 97.8 | 1 | 1 | 1 |  |  |  |  |  |  | PP | 55 |  |  |  | 1 | 1 | 1 |  |  |  |
| CRT-TEST-085 |  | Sepia shield |  |  |  | 0 | No result |  | 1 | 1 | 1 |  |  |  |  |  |  | PA | 11 | 1 | 1 | 1 |  |  |  |  |  |  |
| CRT-TEST-086 | PA | nylon bolting Rings | fragment | whitish | consumer plastic | 1 | PA66 | 99.2 | 1 | 1 | 1 |  |  |  |  |  |  | PA | 97 | 1 | 1 | 1 |  |  |  |  |  |  |
| CRT-TEST-087 |  | Fulmar bill 2 |  |  |  | 0 | PA HTN | 93.2 |  |  |  |  |  |  | 1 | 1 | 1 | PA | 75 | 1 | 1 |  |  |  |  |  |  | 1 |
| CRT-TEST-088 | PP | Food container | fragment | white | consumer plastic | 1 | PP Homo | 98.4 | 1 | 1 | 1 |  |  |  |  |  |  | PP | 97 | 1 | 1 | 1 |  |  |  |  |  |  |
| CRT-TEST-089 |  | fishbones from Fulmar stomach |  |  |  | 0 | Arabic | 88.1 | 1 |  |  |  |  |  |  | 1 | 1 | PA | 51 | 1 | 1 | 1 |  |  |  |  |  |  |
| CRT-TEST-090 | PVC | Cabrion Roof cover | rubber | black | consumer plastic | 1 | PMMA | 94.8 |  |  |  |  |  |  | 1 | 1 | 1 | PS | 13 |  |  |  | 1 | 1 | 1 |  |  |  |
| CRT-TEST-091 |  | Seaweed |  |  |  | 0 | Arabic | 94.1 |  |  |  |  |  |  | 1 | 1 | 1 | PA | 52 | 1 | 1 | 1 |  |  |  |  |  |  |
| CRT-TEST-092 | PP | Flag line | thread | white | consumer plastic | 1 | PP Block | 93.6 | 1 | 1 | 1 |  |  |  |  |  |  | PP | 84 |  | 1 | 1 | 1 |  |  |  |  |  |
| CRT-TEST-093 |  | tomato seed |  |  |  | 0 | Arabic | 95.8 |  |  |  |  |  |  | 1 | 1 | 1 | IONOMER | 68 | 1 | 1 | 1 |  |  |  |  |  |  |
| CRT-TEST-094 | Polyester | sailing band woven | thread | white | consumer plastic | 1 | PET | 96.2 |  |  |  |  |  |  | 1 | 1 | 1 | PET | 72 |  |  | 1 | 1 | 1 |  |  |  |  |
| CRT-TEST-095 | Arabic | Balloon | rubber | green | consumer plastic | 1 | Arabic | 89.8 |  | 1 | 1 | 1 |  |  |  |  |  | PET | 81 |  |  |  | 1 |  |  |  | 1 | 1 |
| CRT-TEST-096 | PVC | PVC coated on Polyester | sheet | white | consumer plastic | 1 | ABS | 89.8 |  | 1 | 1 | 1 |  |  |  |  |  | PVC | 77 |  |  | 1 | 1 | 1 |  |  |  |  |
| CRT-TEST-097 | PET | Packaging container of a lamp | fragment | transparent | consumer plastic | 1 | PET | 97.3 | 1 | 1 | 1 |  |  |  |  |  |  | PET | 96 | 1 | 1 | 1 |  |  |  |  |  |  |
| CRT-TEST-098 | CA | Cigarette filter (used; beach) | other | whitish | from beach | 1 | PVDC | 80.9 |  |  |  | 1 |  |  |  | 1 | 1 | PA | 62 |  |  |  | 1 | 1 | 1 |  |  |  |
| CRT-TEST-099 |  | Bird bone (sternum Woodcock) |  |  |  | 0 | Arabic | 92.1 |  |  |  |  |  |  | 1 | 1 | 1 | PA | 50 | 1 | 1 | 1 |  |  |  |  |  |  |
| CRT-TEST-100 | PA | Almega sportfishing line 0.5mm | thread | transparent | consumer plastic | 1 | PA6 | 98.8 | 1 | 1 | 1 |  |  |  |  |  |  | PA | 84 |  | 1 | 1 | 1 |  |  |  |  |  |
| CRT-TEST-101 | PVC | Stair profile | rubber | brown | consumer plastic | 1 | No result |  |  |  |  | 1 | 1 | 1 |  |  |  | PET | 40 |  |  |  | 1 | 1 | 1 |  |  |  |
| CRT-TEST-102 | PE | Twisted rope | thread | orange | consumer plastic | 1 | LDPE | 91.6 | 1 | 1 | 1 |  |  |  |  |  |  | PE | 97 | 1 | 1 | 1 |  |  |  |  |  |  |
| CRT-TEST-103 |  | fossil shark tooth |  |  |  | 0 | PVDC | 84.5 | 1 |  |  |  |  |  |  | 1 | 1 | PS | 16 | 1 | 1 | 1 |  |  |  |  |  |  |
| CRT-TEST-104 | PA | Silvery white rope | thread | white | consumer plastic | 1 | PET GF | 97.3 |  |  |  |  |  |  | 1 | 1 | 1 | PET | 84 |  |  |  | 1 |  |  |  | 1 | 1 |
| CRT-TEST-105 | ? | Compostable Greenpen Mater-Bi | fragment | green | bio-plastic | 1 | Polyarylate | 78.3 | 1 | 1 |  |  |  |  |  |  | 1 | PA | 87 |  |  |  | 1 |  |  |  | 1 | 1 |
| CRT-TEST-106 |  | Feathershaft Woodcock |  |  |  | 0 | PA HTN | 93.9 | 1 | 1 | 1 |  |  |  |  |  |  | PA | 53 | 1 | 1 | 1 |  |  |  |  |  |  |
| CRT-TEST-107 |  | Seaweed Laminaria |  |  |  | 0 | Arabic | 93.6 |  |  |  |  |  |  | 1 | 1 | 1 | PA | 43 | 1 | 1 | 1 |  |  |  |  |  |  |
| CRT-TEST-108 |  | Small melon seed |  |  |  | 0 | Arabic | 92.9 |  |  |  |  |  |  | 1 | 1 | 1 | CA | 28 | 1 | 1 | 1 |  |  |  |  |  |  |
| CRT-TEST-109 |  | Ray egg skin from Ducth coast |  |  |  | 0 | PA HTN | 89.8 |  |  |  |  |  |  | 1 | 1 | 1 | PA | 62 | 1 | 1 | 1 |  |  |  |  |  |  |
| CRT-TEST-110 |  | Nereis jaw from Fulmar stomach |  |  |  | 0 | PA HTN | 90.6 |  |  |  |  |  |  | 1 | 1 | 1 | PS | 19 | 1 | 1 | 1 |  |  |  |  |  |  |
| CRT-TEST-111 | PVC | Stair profile | rubber | black | consumer plastic | 1 | No result |  |  |  |  | 1 | 1 | 1 |  |  |  | PPO | 18 |  |  |  | 1 | 1 | 1 |  |  |  |
| CRT-TEST-112 | PE | Jar screw cap | fragment | yellow | consumer plastic | 1 | LDPE | 91.4 | 1 | 1 | 1 |  |  |  |  |  |  | PE | 96 | 1 | 1 | 1 |  |  |  |  |  |  |
| CRT-TEST-113 | PP | Rope | thread | brown | consumer plastic | 1 | PP Random | 97.8 | 1 | 1 | 1 |  |  |  |  |  |  | PP | 89 |  | 1 | 1 | 1 |  |  |  |  |  |
| CRT-TEST-114 |  | fish eyelens from Fulmar stomach |  |  |  | 0 | PA66 GF FR | 90.6 |  |  |  |  |  |  | 1 | 1 | 1 | PA | 72 | 1 | 1 |  |  |  |  |  |  | 1 |
| CRT-TEST-115 |  | Wingshield from insect from fulmar stomach |  |  |  | 0 | PA6/66 GF FR | 89.4 | 1 |  |  |  |  |  |  | 1 | 1 | PA | 67 | 1 | 1 | 1 |  |  |  |  |  |  |
| CRT-TEST-116 |  | Melon seeds from Fulmar stomach |  |  |  | 0 | Arabic | 94.2 |  |  |  |  |  |  | 1 | 1 | 1 | PA | 66 | 1 | 1 | 1 |  |  |  |  |  |  |
| CRT-TEST-117 |  | whelk egg from Fulmar stomach |  |  |  | 0 | PA HTN | 92.4 |  |  |  |  |  |  | 1 | 1 | 1 | PA | 63 | 1 | 1 | 1 |  |  |  |  |  |  |
| CRT-TEST-118 |  | squid jaw part from fulmar stomach |  |  |  | 0 | PA6/66 GF FR | 93.8 |  |  |  |  |  |  | 1 | 1 | 1 | POM | 20 | 1 | 1 | 1 |  |  |  |  |  |  |
| CRT-TEST-119 |  | squid eyelens from Fulmar stomach |  |  |  | 0 | PA6 | 88.4 |  |  |  |  |  |  | 1 | 1 | 1 | PA | 74 | 1 | 1 |  |  |  |  |  |  | 1 |
| CRT-TEST-120 | PA | Bolting ring | fragment | white | consumer plastic | 1 | PA6 | 99.5 | 1 | 1 | 1 |  |  |  |  |  |  | PA | 97 | 1 | 1 | 1 |  |  |  |  |  |  |
| CRT-TEST-121 | PVC | Botaflex rubber | rubber | red | consumer plastic | 1 | Arabic | 88.8 |  |  |  | 1 |  |  |  | 1 | 1 | PVC | 73 |  |  | 1 | 1 | 1 |  |  |  |  |
| CRT-TEST-122 | PE | Plastic bag of postal magazine | sheet | transparent | consumer plastic | 1 | LDPE | 95.4 | 1 | 1 | 1 |  |  |  |  |  |  | PE | 92 | 1 | 1 | 1 |  |  |  |  |  |  |
| CRT-TEST-123 | PP | Rope multifibre | thread | orange | consumer plastic | 1 | PP | 97.3 | 1 | 1 | 1 |  |  |  |  |  |  | PP | 81 |  | 1 | 1 | 1 |  |  |  |  |  |
| CRT-TEST-124 | PA | woven safety band textile | thread | white | consumer plastic | 1 | PET | 93.9 |  |  |  |  |  |  | 1 | 1 | 1 | PET | 74 |  |  |  | 1 | 1 |  |  |  | 1 |
| CRT-TEST-125 |  | fishbone from Fulmar stomach |  |  |  | 0 | Arabic | 85.8 | 1 |  |  |  |  |  |  | 1 | 1 | PA | 48 | 1 | 1 | 1 |  |  |  |  |  |  |
| CRT-TEST-126 |  | fish eyelens very small from Fulmar stomach |  |  |  | 0 | Aramid | 91.3 |  |  |  |  |  |  | 1 | 1 | 1 | PS | 22 | 1 | 1 | 1 |  |  |  |  |  |  |
| CRT-TEST-127 |  | Nereis jaw from Fulmar stomach |  |  |  | 0 | PA6/66 GF FR | 91.4 |  |  |  |  |  |  | 1 | 1 | 1 | PA | 66 | 1 | 1 | 1 |  |  |  |  |  |  |
| CRT-TEST-128 |  | otolith Whiting from Fulmar stomach |  |  |  | 0 | No result |  | 1 | 1 | 1 |  |  |  |  |  |  | PA | 32 | 1 | 1 | 1 |  |  |  |  |  |  |
| CRT-TEST-129 | SBS rubber | door stop | rubber | brown | consumer plastic | 1 | SBS | 84.2 |  | 1 | 1 | 1 |  |  |  |  |  | PP | 13 |  |  |  | 1 | 1 | 1 |  |  |  |
| CRT-TEST-130 | SIS rubber | door stop | rubber | white | consumer plastic | 1 | SIS | 87.8 |  | 1 | 1 | 1 |  |  |  |  |  | PVC | 88 |  |  |  | 1 |  |  |  | 1 | 1 |
| CRT-TEST-131 |  | Elastic Natural Rubber band broad | rubber | brown |  | 0 | Arabic | 89 | 1 |  |  |  |  |  |  | 1 | 1 | PVC | 86 | 1 |  |  |  |  |  |  | 1 | 1 |
| CRT-TEST-132 |  | Elastic Natural Rubber band narrow | rubber | brown |  | 0 | Arabic | 88.3 | 1 |  |  |  |  |  |  | 1 | 1 | PET | 87 | 1 |  |  |  |  |  |  | 1 | 1 |
| CRT-TEST-133 | PE | Beamtrawl net | thread | green | consumer plastic | 1 | PVDC | 77.9 |  |  |  | 1 | 1 |  |  |  | 1 | PE | 92 | 1 | 1 | 1 |  |  |  |  |  |  |
| CRT-TEST-134 | 7P0204 | Compostable banana bag; crispy | sheet | transparent | bio-plastic | 1 | No result |  | 1 | 1 | 1 |  |  |  |  |  |  | PMMA | 69 | 1 | 1 | 1 |  |  |  |  |  |  |
| CRT-TEST-135 |  | Manilla natural rope | thread | brown |  | 0 | Arabic | 93.6 |  |  |  |  |  |  | 1 | 1 | 1 | PA | 57 | 1 | 1 | 1 |  |  |  |  |  |  |
| CRT-TEST-136 |  | Parasitic copepod tubing |  |  |  | 0 | PA6/66 GF FR | 89.4 | 1 |  |  |  |  |  |  | 1 | 1 | PA | 73 | 1 | 1 |  |  |  |  |  |  | 1 |
| CRT-TEST-137 |  | Vertebra fish from Fulmar stomach |  |  |  | 0 | Arabic | 80.7 | 1 |  |  |  |  |  |  | 1 | 1 | PA | 56 | 1 | 1 | 1 |  |  |  |  |  |  |
| CRT-TEST-138 | PS | vGansewinkel reference material | fragment | greyish white | raw material | 1 | PS | 97.3 | 1 | 1 | 1 |  |  |  |  |  |  | PS | 94 | 1 | 1 | 1 |  |  |  |  |  |  |
| CRT-TEST-139 | PS | vGansewinkel reference material | fragment | white | raw material | 1 | Urethan | 79.5 |  |  |  | 1 | 1 |  |  |  | 1 | ABS | 93 |  |  |  |  |  |  | 1 | 1 | 1 |
| CRT-TEST-140 | PE | vGansewinkel reference material | fragment | blue | raw material | 1 | LDPE | 91.8 | 1 | 1 | 1 |  |  |  |  |  |  | PE | 97 | 1 | 1 | 1 |  |  |  |  |  |  |
| CRT-TEST-141 | PE | vGansewinkel reference material | fragment | grey | raw material | 1 | LDPE | 92.1 | 1 | 1 | 1 |  |  |  |  |  |  | PE | 91 | 1 | 1 | 1 |  |  |  |  |  |  |
| CRT-TEST-142 | PE | vGansewinkel reference material | fragment | green | raw material | 1 | LDPE | 92.2 | 1 | 1 | 1 |  |  |  |  |  |  | PE | 88 |  | 1 | 1 | 1 |  |  |  |  |  |
| CRT-TEST-143 | PE | vGansewinkel reference material | fragment | black | raw material | 1 | LDPE | 92.4 | 1 | 1 | 1 |  |  |  |  |  |  | PS | 10 |  |  |  | 1 | 1 | 1 |  |  |  |
| CRT-TEST-144 |  | Operculum whelk from Fulmar stomach |  |  |  | 0 | PA6/66 GF FR | 92.4 |  |  |  |  |  |  | 1 | 1 | 1 | PA | 75 | 1 | 1 |  |  |  |  |  |  | 1 |
| CRT-TEST-145 |  | Onion skin from Fulmar stomach |  |  |  | 0 | Arabic | 92.8 |  |  |  |  |  |  | 1 | 1 | 1 | PA | 48 | 1 | 1 | 1 |  |  |  |  |  |  |
| CRT-TEST-146 |  | Fishbone from Fulmar stomach |  |  |  | 0 | Arabic | 87.1 | 1 |  |  |  |  |  |  | 1 | 1 | PA | 58 | 1 | 1 | 1 |  |  |  |  |  |  |
| CRT-TEST-147 | Silica | Silica gel desiccant spherules | other | purple | consumer plastic | 1 | Cellopha | 89.4 |  |  |  | 1 |  |  |  | 1 | 1 | PMMA | 7 | 1 | 1 | 1 |  |  |  |  |  |  |
| CRT-TEST-148 |  | Fishbone from Fulmar stomach |  |  |  | 0 | Arabic | 81.9 | 1 |  |  |  |  |  |  | 1 | 1 | POM | 30 | 1 | 1 | 1 |  |  |  |  |  |  |
| CRT-TEST-149 |  | Ray egg skin from Dutch coast |  |  |  | 0 | PA6/66 GF FR | 88.5 | 1 |  |  |  |  |  |  | 1 | 1 | PA | 20 | 1 | 1 | 1 |  |  |  |  |  |  |
| CRT-TEST-150 | PE | vGansewinkel reference material | fragment | black | raw material | 1 | LDPE | 90.5 | 1 | 1 | 1 |  |  |  |  |  |  | PP | 18 |  |  |  | 1 | 1 | 1 |  |  |  |
| CRT-TEST-151 | Arabic | Balloon rubber | other | mint green | consumer plastic | 1 | Arabic | 78 |  |  | 1 | 1 | 1 |  |  |  |  | PVC | 83 |  |  |  | 1 |  |  |  | 1 | 1 |
| CRT-TEST-152 |  | Seaweed dried; Sacharina |  |  |  | 0 | Arabic | 86.3 | 1 |  |  |  |  |  |  | 1 | 1 | PA | 41 | 1 | 1 | 1 |  |  |  |  |  |  |
| CRT-TEST-153 |  | squid jaw part from Fulmar stomach |  |  |  | 0 | PA6/66 GF FR | 92.4 |  |  |  |  |  |  | 1 | 1 | 1 | PA | 70 | 1 | 1 |  |  |  |  |  |  | 1 |
| CRT-TEST-154 | PE | vGansewinkel reference material | fragment | red | raw material | 1 | LDPE | 91.8 | 1 | 1 | 1 |  |  |  |  |  |  | PE | 98 | 1 | 1 | 1 |  |  |  |  |  |  |
| CRT-TEST-155 | ABS | vGansewinkel reference material | fragment | black | raw material | 1 | No result |  |  |  |  | 1 | 1 | 1 |  |  |  | PS | 14 |  |  |  | 1 | 1 | 1 |  |  |  |
| CRT-TEST-156 |  | Bulbous seed unknown |  |  |  | 0 | Arabic | 92.9 |  |  |  |  |  |  | 1 | 1 | 1 | PA | 53 | 1 | 1 | 1 |  |  |  |  |  |  |
| CRT-TEST-157 |  | Wool; sheep; painted blue |  |  |  | 0 | PA6/66 GF FR | 90.6 |  |  |  |  |  |  | 1 | 1 | 1 | PA | 68 | 1 | 1 | 1 |  |  |  |  |  |  |
| CRT-TEST-158 |  | Seal whisker |  |  |  | 0 | Aramid | 87.6 | 1 |  |  |  |  |  |  | 1 | 1 | PA | 68 | 1 | 1 | 1 |  |  |  |  |  |  |
| CRT-TEST-159 | ABS | vGansewinkel reference material | fragment | grey | raw material | 1 | ABS | 97.2 | 1 | 1 | 1 |  |  |  |  |  |  | ABS | 92 | 1 | 1 | 1 |  |  |  |  |  |  |
| CRT-TEST-160 |  | Bird bill outer layer (keratin) Eiderduck |  |  |  | 0 | Arabic | 90.7 |  |  |  |  |  |  | 1 | 1 | 1 | PA | 70 | 1 | 1 |  |  |  |  |  |  | 1 |
| CRT-TEST-161 | ABS | vGansewinkel reference material | fragment | grey-white | raw material | 1 | ABS | 82.9 |  | 1 | 1 | 1 |  |  |  |  |  | PPO | 78 |  |  |  | 1 | 1 |  |  |  | 1 |
| CRT-TEST-162 |  | Feathershaft Eiderduck |  |  |  | 0 | PA6/66 GF FR | 93.8 |  |  |  |  |  |  | 1 | 1 | 1 | PA | 73 | 1 | 1 |  |  |  |  |  |  | 1 |
| CRT-TEST-163 | ABS | vGansewinkel reference material | fragment | green-transparent | raw material | 1 | ABS | 93.8 | 1 | 1 | 1 |  |  |  |  |  |  | ABS | 94 | 1 | 1 | 1 |  |  |  |  |  |  |
| CRT-TEST-164 | Silica | Silica gel desiccant spherules | other | transparent | consumer plastic | 1 | Arabic | 86 |  |  |  | 1 |  |  |  | 1 | 1 | PMMA | 5 | 1 | 1 | 1 |  |  |  |  |  |  |
| CRT-TEST-165 | ABS | vGansewinkel reference material | fragment | milky-transparent | raw material | 1 | PA612 | 96.5 |  |  |  |  |  |  | 1 | 1 | 1 | PA/ABS | 69 |  |  |  | 1 | 1 | 1 |  |  |  |
| CRT-TEST-166 |  | Bone bird Eiderduck sternum |  |  |  | 0 | Arabic | 89.9 | 1 |  |  |  |  |  |  | 1 | 1 | PA | 55 | 1 | 1 | 1 |  |  |  |  |  |  |
| CRT-TEST-167 |  | Footweb skin Eiderduck |  |  |  | 0 | PA6/66 GF FR | 90 |  |  |  |  |  |  | 1 | 1 | 1 | PA | 67 | 1 | 1 | 1 |  |  |  |  |  |  |
| CRT-TEST-168 | PE | Industrial pellet; beach | pellet | green | from beach | 1 | No result |  |  |  |  | 1 | 1 | 1 |  |  |  | PE | 90 | 1 | 1 | 1 |  |  |  |  |  |  |
| CRT-TEST-169 | PE | Industrial pellet; beach | pellet | transparent | from beach | 1 | No result |  |  |  |  | 1 | 1 | 1 |  |  |  | PE | 94 | 1 | 1 | 1 |  |  |  |  |  |  |
| CRT-TEST-170 | PE | Industrial pellet; beach | pellet | white | from beach | 1 | Silicon | 80.2 |  |  |  | 1 |  |  |  | 1 | 1 | PE | 96 | 1 | 1 | 1 |  |  |  |  |  |  |
| CRT-TEST-171 |  | Insect wing of fly |  |  |  | 0 | Aramid | 90.1 |  |  |  |  |  |  | 1 | 1 | 1 | ABS | 24 | 1 | 1 | 1 |  |  |  |  |  |  |
| CRT-TEST-172 | NBR rubber | Industrial pellet; beach | pellet | black | from beach | 1 | NBR | 81.1 |  | 1 | 1 | 1 |  |  |  |  |  | PS | 30 |  |  |  | 1 | 1 | 1 |  |  |  |
| CRT-TEST-173 |  | Seaweed Ulva |  |  |  | 0 | Arabic | 92.2 |  |  |  |  |  |  | 1 | 1 | 1 | PA | 46 | 1 | 1 | 1 |  |  |  |  |  |  |
| CRT-TEST-174 | PP | Industrial pellet; beach | pellet | white | from beach | 1 | No result |  |  |  |  | 1 | 1 | 1 |  |  |  | PP | 85 |  | 1 | 1 | 1 |  |  |  |  |  |
| CRT-TEST-175 |  | Barnacle parts |  |  |  | 0 | Melamin | 77.9 | 1 | 1 |  |  |  |  |  |  | 1 | PA | 23 | 1 | 1 | 1 |  |  |  |  |  |  |
| CRT-TEST-176 | PE | Plastic bag heavy sheet | sheet | transparent | consumer plastic | 1 | LDPE | 95.8 | 1 | 1 | 1 |  |  |  |  |  |  | PE | 95 | 1 | 1 | 1 |  |  |  |  |  |  |
| CRT-TEST-177 | PS | Black underside of CD box | fragment | black | consumer plastic | 1 | PS | 98.6 | 1 | 1 | 1 |  |  |  |  |  |  | PS | 8 |  |  |  | 1 | 1 | 1 |  |  |  |
| CRT-TEST-178 | PS | Transparent upperside of CD box | fragment | transparent | consumer plastic | 1 | PS | 98 | 1 | 1 | 1 |  |  |  |  |  |  | PS | 95 | 1 | 1 | 1 |  |  |  |  |  |  |
| CRT-TEST-179 |  | Crab carapax |  |  |  | 0 | Melamin | 83.1 | 1 |  |  |  |  |  |  | 1 | 1 | PA | 38 | 1 | 1 | 1 |  |  |  |  |  |  |
| CRT-TEST-180 |  | Clay-pellet hydrokorrel |  |  |  | 0 | Silicon | 84.6 | 1 |  |  |  |  |  |  | 1 | 1 | PS | 17 | 1 | 1 | 1 |  |  |  |  |  |  |
| CRT-TEST-181 | PE | Soft-foamed sheet for packaging | foam | white | consumer plastic | 1 | LDPE | 95.7 | 1 | 1 | 1 |  |  |  |  |  |  | PE | 58 |  |  |  | 1 | 1 | 1 |  |  |  |
| CRT-TEST-182 | PE | Bubble wrap | sheet | transparent | consumer plastic | 1 | LDPE | 93.9 | 1 | 1 | 1 |  |  |  |  |  |  | PE | 86 |  | 1 | 1 | 1 |  |  |  |  |  |
| CRT-TEST-183 |  | Stone from Fulmar stomach |  |  |  | 0 | No result |  | 1 | 1 | 1 |  |  |  |  |  |  | PA | 22 | 1 | 1 | 1 |  |  |  |  |  |  |
| CRT-TEST-184 | PE | soft airgun bullet | other | yellow | from beach | 1 | Ionomer | 84.7 |  |  |  | 1 |  |  |  | 1 | 1 | PE | 98 | 1 | 1 | 1 |  |  |  |  |  |  |
| CRT-TEST-185 | PS | Piepschuim - Polysterene foam cell | foam | white | consumer plastic | 1 | HIPS | 94.3 | 1 | 1 | 1 |  |  |  |  |  |  | ABS | 70 |  |  |  | 1 | 1 |  |  |  | 1 |
| CRT-TEST-186 | PE | cap of jerrycan from beach | fragment | red | from beach | 1 | LDPE | 91.5 | 1 | 1 | 1 |  |  |  |  |  |  | PE | 98 | 1 | 1 | 1 |  |  |  |  |  |  |
| CRT-TEST-187 | PE | Bottle cap - from beach | fragment | blue dark | from beach | 1 | Ionomer | 89.1 |  |  |  | 1 |  |  |  | 1 | 1 | PE | 97 | 1 | 1 | 1 |  |  |  |  |  |  |
| CRT-TEST-188 |  | chemical? palmfat? | other |  | paraffine | 1 | LDPE | 91.8 |  |  |  |  |  |  | 1 | 1 | 1 | PE | 91 |  |  |  |  |  |  | 1 | 1 | 1 |
| CRT-TEST-189 |  | seaweed blaasjeswier |  |  |  | 0 | Arabic | 95.7 |  |  |  |  |  |  | 1 | 1 | 1 | PI | 7 | 1 | 1 | 1 |  |  |  |  |  |  |
| CRT-TEST-190 | PE | Water bottle cap; beach | fragment | blue | from beach | 1 | LDPE | 93.8 | 1 | 1 | 1 |  |  |  |  |  |  | PE | 98 | 1 | 1 | 1 |  |  |  |  |  |  |
| CRT-TEST-191 | PVC | heavy type of bag in which a shirt was packed | sheet | transparent | consumer plastic | 1 | PVC | 86.6 |  | 1 | 1 | 1 |  |  |  |  |  | PVC | 96 | 1 | 1 | 1 |  |  |  |  |  |  |
| CRT-TEST-192 |  | Shellfish Oyster |  |  |  | 0 | No result |  | 1 | 1 | 1 |  |  |  |  |  |  | PA | 16 | 1 | 1 | 1 |  |  |  |  |  |  |
| CRT-TEST-193 | PP | plant pot; beach | fragment | brown | from beach | 1 | PP | 95 | 1 | 1 | 1 |  |  |  |  |  |  | PP | 95 | 1 | 1 | 1 |  |  |  |  |  |  |
| CRT-TEST-194 | PE | plug on airvalve of jerrycan; beach | fragment | transparent | from beach | 1 | LDPE | 95.9 | 1 | 1 | 1 |  |  |  |  |  |  | PE | 98 | 1 | 1 | 1 |  |  |  |  |  |  |
| CRT-TEST-195 |  | Toenail Eiderduck |  |  |  | 0 | PA66 GF FR | 87.6 | 1 |  |  |  |  |  |  | 1 | 1 | PA | 67 | 1 | 1 | 1 |  |  |  |  |  |  |
| CRT-TEST-196 | PET | softdrink bottle; beach | fragment | transparent | from beach | 1 | PET | 96.2 | 1 | 1 | 1 |  |  |  |  |  |  | PET | 97 | 1 | 1 | 1 |  |  |  |  |  |  |
| CRT-TEST-197 | PET | mineral water bottle; beach | fragment | bluish transparent | from beach | 1 | PET | 96.7 | 1 | 1 | 1 |  |  |  |  |  |  | PET | 97 | 1 | 1 | 1 |  |  |  |  |  |  |
| CRT-TEST-198 |  | chemical? Paraffin? | other | white | paraffine | 1 | LDPE | 93.9 |  |  |  |  |  |  | 1 | 1 | 1 | PE | 82 | 1 |  |  |  |  |  |  | 1 | 1 |
| CRT-TEST-199 | PP | ice box | fragment | white | consumer plastic | 1 | PP | 97.3 | 1 | 1 | 1 |  |  |  |  |  |  | PP | 96 | 1 | 1 | 1 |  |  |  |  |  |  |
| CRT-TEST-200 |  | Squid eyelens from Fulmar stomach |  |  |  | 0 | PA6/66 GF FR | 92.4 |  |  |  |  |  |  | 1 | 1 | 1 | PA | 76 | 1 | 1 |  |  |  |  |  |  | 1 |

## Online Supplement Table 2 Polymer type abbreviations

| Abbreviation | Name |
| --- | --- |
| ABS* | Acrylonitrile butadiene styrene |
| CA* | Cellulose acetate |
| EMA | Copolymer of polyethylene |
| EST* | Elastomer |
| EVA* | Ethylene-vinyl acetate |
| HDPE | High density polyethylene |
| HIPS | High impact polystyrene |
| ION* | Ionmer |
| LDPE | Low density polyethylene |
| NABS* | Nylon/ABSblend |
| PA* | Nylon (polyamide) |
| PB* | Polybutylene |
| PBT* | Polybutylene terephthalate |
| PC* | Polycarbonate |
| PE* | Polyethylene |
| PET* | Polyethylene terephthalate |
| PETG* | Polyethylene terephthalate glycol |
| PI* | Polyimide |
| PMMA* | Polymethyl metacrylate |
| PMP* | Polymethyl pentane |
| POM* | Acetal (Polyoxymethylene) |
| PP* | Polypropylene |
| PPO* | Polyphenylene oxide |
| PPS* | Polyphenolyne sulfide |
| PS* | Polystyrene |
| PSO* | Polysulfone |
| PTT* | Polytrimethylene terephthalate |
| PUR* | Polyurethane |
| PVC* | Polyvinyl chloride |
| PVCD | Polyvinylidene chloride |
| SAN* | Styrene acrylonitrile |
| STP* | Styrenic terpolymer |
| TPV* | Thermoplastic elastomer |

*Integrated in Phazir NIR library

## Online Supplement Table 3: Details on plastic categories and polymer types found in this study

**Table OS 3.1. Details of plastic categories found in seabirds.** Per species, region and decennia, the number and percentage of each plastic category are given.

**Table OS 3.2a. Plastic categories and polymer types in all birds.** Number and mass of plastic categories with associated percentages. Included are plastics ingested by all birds (all species, all years, all locations).

**Table OS 3.2b. Plastic categories and polymer types in northern fulmars.** Number and mass of plastic categories with associated percentages. Included are plastics ingested by all northern fulmars (all years, all northern hemisphere locations).

**Table OS 3.3a. Temporal comparison.** Number and mass of plastics ingested by northern fulmars from the Netherlands during three decennia with associated percentages.

**Table OS 3.3b.** **Temporal comparison.** Number and mass per polymer type of plastics ingested by northern fulmars from the Netherlands during three decennia with associated percentages. Match score threshold is set at >80%.

**Table OS 3.4b.** **Regional comparison.** Number and mass per polymer type of plastics ingested by northern fulmars from the Netherlands, the Faroe Islands, Iceland and Svalbard with associated percentages. Match score threshold is set at >080%.

**Table OS 3.4a. Regional comparison.** Number and mass of plastics ingested by northern fulmars from the Netherlands (2010-2019), the Faroe Islands, Iceland and Svalbard with associated percentages.

**Table OS 3.5a. Global comparison.** Number of plastics ingested by seabird species from Antarctica, with associated percentages.

**Table OS 3.5b.** **Global comparison.** Number and mass per polymer type of plastics ingested by seabird species from Antarctica, with associated percentages. Match score threshold is set at >80%.

## Online Supplement Table 4. Details on polymer identification methods using infrared spectroscopy and match score thresholds applied in different studies.

Search ended in March 2020.

| **Group** | **Threshold level >%** | **References** |
| --- | --- | --- |
| Seabirds | 80% | Leopold et al. 2019; this study* |
|  | 75% | Amélineau et al. 2016; Le Guen et al. 2020 |
|  | 60-85% | Avery-Gomm et al. 2016 |
|  | No details | Álvarez et al. 2018; Bessa et al. 2019; Lenzi et al. 2016; Nicastro et al. 2018; Yamashita et al. 2011*; Zhu et al. 2019a |
| Marine mammals | 80% | Moore et al. 2019; Van Franeker et al. 2018 |
|  | 70% | Hudak & Sette 2019; Nelms et al. 2019; Caron et al. 2018 |
|  | 60% | Lusher et al. 2015 |
|  | No details | Besseling et al. 2015; Bravo Rebolledo & van Franeker 2015; Eriksson & Burton 2003; Perez-Venegas et al. 2020; Zhu et al. 2019b |
| Turtles | No details | Pham et al. 2017; Rizzi et al. 2019 |
| Marine fish | 93% | Savoca et al. 2019 |
|  | 85% | Bessa et al. 2018; Capillo et al. 2020; Ory et al. 2017 |
|  | 80% | Bernardini et al. 2018; Digka et al. 2018; Garcia-Garin et al. 2019; Karthik et al. 2018 |
|  | 70% | Akoueson et al. 2020; Alomar et al. 2017; Bour et al. 2018; Goswami et al. 2020; Lefebvre et al. 2019; Morgana et al. 2018; Nelms et al. 2018; Ogonowski et al. 2019; Ory et al. 2018; Su et al. 2019; Tanaka & Takada 2016; Zhang et al. 2019 |
|  | 75% | Kühn et al. 2020 |
|  | 60% | Avio et al. 2015; Avio et al. 2020; Bucol et al. 2020; Kroon et al. 2018; Lusher et al. 2013; Markic et al. 2018 |
|  | No details | Al-Salem et al. 2020; Alomar & Deudero 2017; Baalkhuyur et al. 2020; Bianchi et al. 2020; Bråte et al. 2016; Cannon et al. 2016; Chagnon et al. 2018; Chan et al. 2019; Cheung et al. 2018; Compa et al. 2018; Fernández & Anastasopoulou 2019; Foekema et al. 2013; Giani et al. 2019; Güven et al. 2017; Halstead et al. 2018; Hermsen et al. 2017; Herrera et al. 2019; Jabeen et al. 2017; Karlsson et al. 2017; Karuppasamy et al. 2020; Kühn et al. 2018; Kumar et al. 2018; McGoran et al. 2017; Murphy et al. 2017; Neves et al. 2015; Pegado et al. 2018; Pellini et al. 2018; Pozo et al. 2019; Renzi et al. 2019; Rummel et al. 2016; Sbrana et al. 2020; Steer et al. 2017; Welden et al. 2018; Wesch et al. 2016; Wieczorek et al. 2018; Zhu et al. 2019c |
| *NIR was used | | |

## Online Supplement References

Akoueson F, Sheldon LM, Danopoulos E, Morris S, Hotten J, Chapman E, Li J, Rotchell JM (2020) A preliminary analysis of microplastics in edible versus non-edible tissues from seafood samples. Environmental Pollution 263: 114452 doi <https://doi.org/10.1016/j.envpol.2020.114452>

Al-Salem SM, Uddin S, Lyons B (2020) Evidence of microplastics (MP) in gut content of major consumed marine fish species in the State of Kuwait (of the Arabian/Persian Gulf). Marine Pollution Bulletin 154: 111052 doi <https://doi.org/10.1016/j.marpolbul.2020.111052>

Alomar C, Deudero S (2017) Evidence of microplastic ingestion in the shark *Galeus melastomus* Rafinesque, 1810 in the continental shelf off the western Mediterranean Sea. Environmental Pollution 223: 223-229 doi <http://dx.doi.org/10.1016/j.envpol.2017.01.015>

Alomar C, Sureda A, Capó X, Guijarro B, Tejada S, Deudero S (2017) Microplastic ingestion by *Mullus surmuletus* Linnaeus, 1758 fish and its potential for causing oxidative stress. Environmental Research 159: 135-142 doi <http://dx.doi.org/10.1016/j.envres.2017.07.043>

Álvarez G, Barros Á, Velando A (2018) The use of European shag pellets as indicators of microplastic fibers in the marine environment. Marine Pollution Bulletin 137: 444-448 doi <https://doi.org/10.1016/j.marpolbul.2018.10.050>

Amélineau F, Bonnet D, Heitz O, Mortreux V, Harding AM, Karnovsky N, Walkusz W, Fort J, Gremillet D (2016) Microplastic pollution in the Greenland Sea: Background levels and selective contamination of planktivorous diving seabirds. Environmental Pollution 219: 1131-1139 doi <http://dx.doi.org/10.1016/j.envpol.2016.09.017>

Avery-Gomm S, Valliant M, Schacter CR, Robbins KF, Liboiron M, Daoust P-Y, Rios LM, Jones IL (2016) A study of wrecked Dovekies (*Alle alle*) in the western North Atlantic highlights the importance of using standardized methods to quantify plastic ingestion. Marine Pollution Bulletin 113: 75-80 doi <http://dx.doi.org/10.1016/j.marpolbul.2016.08.062>

Avio CG, Gorbi S, Regoli F (2015) Experimental development of a new protocol for extraction and characterization of microplastics in fish tissues: first observations in commercial species from Adriatic Sea. Marine Environmental Research 111: 18-26 doi <http://dx.doi.org/10.1016/j.marenvres.2015.06.014>

Avio CG, Pittura L, d’Errico G, Abel S, Amorello S, Marino G, Gorbi S, Regoli F (2020) Distribution and characterization of microplastic particles and textile microfibers in Adriatic food webs: General insights for biomonitoring strategies. Environmental Pollution 258: 113766 doi <https://doi.org/10.1016/j.envpol.2019.113766>

Baalkhuyur FM, Qurban MA, Panickan P, Duarte CM (2020) Microplastics in fishes of commercial and ecological importance from the Western Arabian Gulf. Marine Pollution Bulletin 152: 110920 doi <https://doi.org/10.1016/j.marpolbul.2020.110920>

Bernardini I, Garibaldi F, Canesi L, Fossi MC, Baini M (2018) First data on plastic ingestion by blue sharks (*Prionace glauca*) from the Ligurian Sea (North-Western Mediterranean Sea). Marine Pollution Bulletin 135: 303-310 doi <https://doi.org/10.1016/j.marpolbul.2018.07.022>

Bessa F, Barría P, Neto JM, Frias JP, Otero V, Sobral P, Marques JC (2018) Microplastics in Juvenile Commercial Fish from an Estuarine Environment, Proceedings of the International Conference on Microplastic Pollution in the Mediterranean Sea, pp 131-135

Bessa F, Ratcliffe N, Otero V, Sobral P, Marques JC, Waluda CM, Trathan PN, Xavier JC (2019) Microplastics in gentoo penguins from the Antarctic region. Scientific Reports 9: 14191 doi 10.1038/s41598-019-50621-2

Besseling E, Foekema EM, Van Franeker JA, Leopold MF, Kühn S, Bravo Rebolledo EL, Heße E, Mielke L, IJzer J, Kamminga P (2015) Microplastic in a macro filter feeder: Humpback whale *Megaptera novaeangliae*. Marine Pollution Bulletin 95: 248-252 doi <http://dx.doi.org/10.1016/j.marpolbul.2015.04.007>

Bianchi J, Valente T, Scacco U, Cimmaruta R, Sbrana A, Silvestri C, Matiddi M (2020) Food preference determines the best suitable digestion protocol for analysing microplastic ingestion by fish. Marine Pollution Bulletin 154: 111050 doi <https://doi.org/10.1016/j.marpolbul.2020.111050>

Bour A, Avio CG, Gorbi S, Regoli F, Hylland K (2018) Presence of microplastics in benthic and epibenthic organisms: Influence of habitat, feeding mode and trophic level. Environmental Pollution 243: 1217-1225 doi <https://doi.org/10.1016/j.envpol.2018.09.115>

Bråte ILN, Eidsvoll DP, Steindal CC, Thomas KV (2016) Plastic ingestion by Atlantic cod (*Gadus morhua*) from the Norwegian coast. Marine Pollution Bulletin 112: 105-110 doi <http://dx.doi.org/10.1016/j.marpolbul.2016.08.034>

Bravo Rebolledo EL, van Franeker J (2015) Impact of marine debris on Antarctic fur seals *Arctocephalus gazella* at Cape Shirreff: diet dependent ingestion and entanglement - Preliminary Results. IMARES, The Netherlands, pp 6

Bucol LA, Romano EF, Cabcaban SM, Siplon LMD, Madrid GC, Bucol AA, Polidoro B (2020) Microplastics in marine sediments and rabbitfish (Siganus fuscescens) from selected coastal areas of Negros Oriental, Philippines. Marine Pollution Bulletin 150: 110685 doi <https://doi.org/10.1016/j.marpolbul.2019.110685>

Cannon SME, Lavers JL, Figueiredo B (2016) Plastic ingestion by fish in the Southern Hemisphere: A baseline study and review of methods. Marine Pollution Bulletin 107: 286-291 doi <http://dx.doi.org/10.1016/j.marpolbul.2016.03.057>

Capillo G, Savoca S, Panarello G, Mancuso M, Branca C, Romano V, D'Angelo G, Bottari T, Spanò N (2020) Quali-quantitative analysis of plastics and synthetic microfibers found in demersal species from Southern Tyrrhenian Sea (Central Mediterranean). Marine Pollution Bulletin 150: 110596 doi <https://doi.org/10.1016/j.marpolbul.2019.110596>

Caron AGM, Thomas CR, Berry KLE, Motti CA, Ariel E, Brodie JE (2018) Ingestion of microplastic debris by green sea turtles (*Chelonia mydas*) in the Great Barrier Reef: Validation of a sequential extraction protocol. Marine Pollution Bulletin 127: 743-751 doi <https://doi.org/10.1016/j.marpolbul.2017.12.062>

Chagnon C, Thiel M, Antunes J, Ferreira JL, Sobral P, Ory NC (2018) Plastic ingestion and trophic transfer between Easter Island flying fish (*Cheilopogon rapanouiensis*) and yellowfin tuna (*Thunnus albacares*) from Rapa Nui (Easter Island). Environmental Pollution 243: 127-133 doi <https://doi.org/10.1016/j.envpol.2018.08.042>

Chan HSH, Dingle C, Not C (2019) Evidence for non-selective ingestion of microplastic in demersal fish. Marine Pollution Bulletin 149: 110523 doi <https://doi.org/10.1016/j.marpolbul.2019.110523>

Cheung LT, Lui CY, Fok L (2018) Microplastic Contamination of Wild and Captive Flathead Grey Mullet (*Mugil cephalus*). International Journal of Environmental Research and Public Health 15: 597 doi <https://doi.org/10.3390/ijerph15040597>

Compa M, Ventero A, Iglesias M, Deudero S (2018) Ingestion of microplastics and natural fibres in *Sardina pilchardus* (Walbaum, 1792) and *Engraulis encrasicolus* (Linnaeus, 1758) along the Spanish Mediterranean coast. Marine Pollution Bulletin 128: 89-96 doi <https://doi.org/10.1016/j.marpolbul.2018.01.009>

Digka N, Tsangaris C, Torre M, Anastasopoulou A, Zeri C (2018) Microplastics in mussels and fish from the Northern Ionian Sea. Marine Pollution Bulletin 135: 30-40 doi <https://doi.org/10.1016/j.marpolbul.2018.06.063>

Eriksson C, Burton H (2003) Origins and Biological Accumulation of Small Plastic Particles in Fur Seals from Macquarie Island. AMBIO: A Journal of the Human Environment 32: 380-384 doi <http://dx.doi.org/10.1579/0044-7447-32.6.380>

Fernández C, Anastasopoulou A (2019) Plastic ingestion by blue shark Prionace glauca in the South Pacific Ocean (south of the Peruvian Sea). Marine Pollution Bulletin 149: 110501 doi <https://doi.org/10.1016/j.marpolbul.2019.110501>

Foekema EM, De Gruijter C, Mergia MT, van Franeker JA, Murk AJ, Koelmans AA (2013) Plastic in North Sea Fish. Environmental Science & Technology 47: 8818-8824 doi <https://doi.org/10.1021/es400931b>

Garcia-Garin O, Vighi M, Aguilar A, Tsangaris C, Digka N, Kaberi H, Borrell A (2019) Boops boops as a bioindicator of microplastic pollution along the Spanish Catalan coast. Marine Pollution Bulletin 149: 110648 doi <https://doi.org/10.1016/j.marpolbul.2019.110648>

Giani D, Baini M, Galli M, Casini S, Fossi MC (2019) Microplastics occurrence in edible fish species (*Mullus barbatus* and *Merluccius merluccius*) collected in three different geographical sub-areas of the Mediterranean Sea. Marine Pollution Bulletin 140: 129-137 doi <https://doi.org/10.1016/j.marpolbul.2019.01.005>

Goswami P, Vinithkumar NV, Dharani G (2020) First evidence of microplastics bioaccumulation by marine organisms in the Port Blair Bay, Andaman Islands. Marine Pollution Bulletin 155: 111163 doi <https://doi.org/10.1016/j.marpolbul.2020.111163>

Güven O, Gökdağ K, Jovanović B, Kıdeyş AE (2017) Microplastic litter composition of the Turkish territorial waters of the Mediterranean Sea, and its occurrence in the gastrointestinal tract of fish. Environmental Pollution 223: 286-294 doi <http://dx.doi.org/10.1016/j.envpol.2017.01.025>

Halstead JE, Smith JA, Carter EA, Lay PA, Johnston EL (2018) Assessment tools for microplastics and natural fibres ingested by fish in an urbanised estuary. Environmental Pollution 234: 552-561 doi <https://doi.org/10.1016/j.envpol.2017.11.085>

Hermsen E, Pompe R, Besseling E, Koelmans AA (2017) Detection of low numbers of microplastics in North Sea fish using strict quality assurance criteria. Marine Pollution Bulletin 122: 253-258 doi <http://dx.doi.org/10.1016/j.marpolbul.2017.06.051>

Herrera A, Ŝtindlová A, Martínez I, Rapp J, Romero-Kutzner V, Samper MD, Montoto T, Aguiar-González B, Packard T, Gómez M (2019) Microplastic ingestion by Atlantic chub mackerel (*Scomber colias*) in the Canary Islands coast. Marine Pollution Bulletin 139: 127-135 doi <https://doi.org/10.1016/j.marpolbul.2018.12.022>

Hudak CA, Sette L (2019) Opportunistic detection of anthropogenic micro debris in harbor seal (Phoca vitulina vitulina) and gray seal (Halichoerus grypus atlantica) fecal samples from haul-outs in southeastern Massachusetts, USA. Marine Pollution Bulletin 145: 390-395 doi <https://doi.org/10.1016/j.marpolbul.2019.06.020>

Jabeen K, Su L, Li J, Yang D, Tong C, Mu J, Shi H (2017) Microplastics and mesoplastics in fish from coastal and fresh waters of China. Environmental Pollution 221: 141-149 doi <http://dx.doi.org/10.1016/j.envpol.2016.11.055>

Karlsson TM, Vethaak AD, Almroth BC, Ariese F, van Velzen M, Hassellöv M, Leslie HA (2017) Screening for microplastics in sediment, water, marine invertebrates and fish: Method development and microplastic accumulation. Marine Pollution Bulletin 122: 403-408 doi <http://dx.doi.org/10.1016/j.marpolbul.2017.06.081>

Karthik R, Robin RS, Purvaja R, Ganguly D, Anandavelu I, Raghuraman R, Hariharan G, Ramakrishna A, Ramesh R (2018) Microplastics along the beaches of southeast coast of India. Science of The Total Environment 645: 1388-1399 doi <https://doi.org/10.1016/j.scitotenv.2018.07.242>

Karuppasamy PK, Ravi A, Vasudevan L, Elangovan MP, Dyana Mary P, Vincent SGT, Palanisami T (2020) Baseline survey of micro and mesoplastics in the gastro-intestinal tract of commercial fish from Southeast coast of the Bay of Bengal. Marine Pollution Bulletin 153: 110974 doi <https://doi.org/10.1016/j.marpolbul.2020.110974>

Kroon FJ, Motti CE, Jensen LH, Berry KL (2018) Classification of marine microdebris: A review and case study on fish from the Great Barrier Reef, Australia. Scientific Reports 8: 16422 doi <http://dx.doi.org/10.1038/s41598-018-34590-6>

Kühn S, Schaafsma FL, van Werven B, Flores H, Bergmann M, Egelkraut-Holtus M, Tekman MB, van Franeker JA (2018) Plastic ingestion by juvenile polar cod (*Boreogadus saida*) in the Arctic Ocean. Polar Biology 41: 1269–1278 doi <http://dx.doi.org/10.1007/s00300-018-2283-8>

Kühn S, van Franeker JA, O’Donoghue AM, Swiers A, Starkenburg M, van Werven B, Foekema E, Hermsen E, Egelkraut-Holtus M, Lindeboom H (2020) Details of plastic ingestion and fibre contamination in North Sea fishes. Environmental Pollution 257: 113569 doi <https://doi.org/10.1016/j.envpol.2019.113569>

Kumar VE, Ravikumar G, Jeyasanta KI (2018) Occurrence of microplastics in fishes from two landing sites in Tuticorin, South east coast of India. Marine Pollution Bulletin 135: 889-894 doi <https://doi.org/10.1016/j.marpolbul.2018.08.023>

Le Guen C, Suaria G, Sherley RB, Ryan PG, Aliani S, Boehme L, Brierley AS (2020) Microplastic study reveals the presence of natural and synthetic fibres in the diet of King Penguins (Aptenodytes patagonicus) foraging from South Georgia. Environment International 134: 105303 doi <https://doi.org/10.1016/j.envint.2019.105303>

Lefebvre C, Saraux C, Heitz O, Nowaczyk A, Bonnet D (2019) Microplastics FTIR characterisation and distribution in the water column and digestive tracts of small pelagic fish in the Gulf of Lions. Marine Pollution Bulletin 142: 510-519 doi <https://doi.org/10.1016/j.marpolbul.2019.03.025>

Lenzi J, Burgues MF, Carrizo D, Machin E, Teixeira-de Mello F (2016) Plastic ingestion by a generalist seabird on the coast of Uruguay. Marine Pollution Bulletin 107: 71-76 doi <http://dx.doi.org/10.1016/j.marpolbul.2016.04.016>

Leopold MF, Kik M, van Tulden P, van Franeker JA, Kühn S, Rijks J (2019) De Zoe en de zeekoet. Een onderzoek naar de doodsoorzaak en de herkomst van de zeekoeten die massaal strandden op de Nederlandse kust in januari en februari 2019. Wageningen Marine Research, Den Helder, The Netherlands, pp 59 doi <https://doi.org/10.18174/472854>

Lusher A, McHugh M, Thompson R (2013) Occurrence of microplastics in the gastrointestinal tract of pelagic and demersal fish from the English Channel. Marine Pollution Bulletin 67: 94-99 doi <http://dx.doi.org/10.1016/j.marpolbul.2012.11.028>

Lusher AL, Hernandez-Milian G, O'Brien J, Berrow S, O'Connor I, Officer R (2015) Microplastic and macroplastic ingestion by a deep diving, oceanic cetacean: The True's beaked whale *Mesoplodon mirus*. Environmental Pollution 199: 185-191 doi <http://dx.doi.org/10.1016/j.envpol.2015.01.023>

Markic A, Niemand C, Bridson JH, Mazouni-Gaertner N, Gaertner J-C, Eriksen M, Bowen M (2018) Double trouble in the South Pacific subtropical gyre: Increased plastic ingestion by fish in the oceanic accumulation zone. Marine Pollution Bulletin 136: 547-564 doi <https://doi.org/10.1016/j.marpolbul.2018.09.031>

McGoran A, Clark P, Morritt D (2017) Presence of microplastic in the digestive tracts of European flounder, *Platichthys flesus,* and European smelt, *Osmerus eperlanus*, from the River Thames. Environmental Pollution 220: 744-751 doi <http://dx.doi.org/10.1016/j.envpol.2016.09.078>

Moore RC, Loseto L, Noel M, Etemadifar A, Brewster JD, MacPhee S, Bendell L, Ross PS (2019) Microplastics in beluga whales (Delphinapterus leucas) from the Eastern Beaufort Sea. Marine Pollution Bulletin: 110723 doi <https://doi.org/10.1016/j.marpolbul.2019.110723>

Morgana S, Ghigliotti L, Estévez-Calvar N, Stifanese R, Wieckzorek A, Doyle T, Christiansen JS, Faimali M, Garaventa F (2018) Microplastics in the Arctic: A case study with sub-surface water and fish samples off Northeast Greenland. Environmental Pollution 242: 1078-1086 doi <https://doi.org/10.1016/j.envpol.2018.08.001>

Murphy F, Russell M, Ewins C, Quinn B (2017) The uptake of macroplastic & microplastic by demersal & pelagic fish in the Northeast Atlantic around Scotland. Marine Pollution Bulletin 122: 353-359 doi <http://dx.doi.org/10.1016/j.marpolbul.2017.06.073>

Nelms S, Barnett J, Brownlow A, Davison N, Deaville R, Galloway T, Lindeque P, Santillo D, Godley B (2019) Microplastics in marine mammals stranded around the British coast: ubiquitous but transitory? Scientific Reports 9: 1075 doi <https://doi.org/10.1038/s41598-018-37428-3>

Nelms SE, Galloway TS, Godley BJ, Jarvis DS, Lindeque PK (2018) Investigating microplastic trophic transfer in marine top predators. Environmental Pollution 238: 999-1007 doi <https://doi.org/10.1016/j.envpol.2018.02.016>

Neves D, Sobral P, Ferreira JL, Pereira T (2015) Ingestion of microplastics by commercial fish off the Portuguese coast. Marine Pollution Bulletin 101: 119-126 doi <http://dx.doi.org/10.1016/j.marpolbul.2015.11.008>

Nicastro KR, Savio RL, McQuaid CD, Madeira P, Valbusa U, Azevedo F, Casero M, Lourenço C, Zardi GI (2018) Plastic ingestion in aquatic-associated bird species in southern Portugal. Marine Pollution Bulletin 126: 413-418 doi <https://doi.org/10.1016/j.marpolbul.2017.11.050>

Ogonowski M, Wenman V, Barth A, Hamacher-Barth E, Danielsson S, Gorokhova E (2019) Microplastic Intake, Its Biotic Drivers, and Hydrophobic Organic Contaminant Levels in the Baltic Herring. Frontiers in Environmental Science 7 doi 10.3389/fenvs.2019.00134

Ory N, Chagnon C, Felix F, Fernández C, Ferreira JL, Gallardo C, Ordóñez OG, Henostroza A, Laaz E, Mizraji R (2018) Low prevalence of microplastic contamination in planktivorous fish species from the southeast Pacific Ocean. Marine Pollution Bulletin 127: 211-216 doi <https://doi.org/10.1016/j.marpolbul.2017.12.016>

Ory NC, Sobral P, Ferreira JL, Thiel M (2017) Amberstripe scad *Decapterus muroadsi* (Carangidae) fish ingest blue microplastics resembling their copepod prey along the coast of Rapa Nui (Easter Island) in the South Pacific subtropical gyre. Science of The Total Environment 586: 430-437 doi <http://dx.doi.org/10.1016/j.scitotenv.2017.01.175>

Pegado TdSeS, Schmid K, Winemiller KO, Chelazzi D, Cincinelli A, Dei L, Giarrizzo T (2018) First evidence of microplastic ingestion by fishes from the Amazon River estuary. Marine Pollution Bulletin 133: 814-821 doi <https://doi.org/10.1016/j.marpolbul.2018.06.035>

Pellini G, Gomiero A, Fortibuoni T, Ferrà C, Grati F, Tassetti N, Polidori P, Fabi G, Scarcella G (2018) Characterization of microplastic litter in the gastrointestinal tract of *Solea solea* from the Adriatic Sea. Environmental Pollution 234: 943-952 doi <https://doi.org/10.1016/j.envpol.2017.12.038>

Perez-Venegas DJ, Toro-Valdivieso C, Ayala F, Brito B, Iturra L, Arriagada M, Seguel M, Barrios C, Sepúlveda M, Oliva D, Cárdenas-Alayza S, Urbina MA, Jorquera A, Castro-Nallar E, Galbán-Malagón C (2020) Monitoring the occurrence of microplastic ingestion in Otariids along the Peruvian and Chilean coasts. Marine Pollution Bulletin 153: 110966 doi <https://doi.org/10.1016/j.marpolbul.2020.110966>

Pham CK, Rodríguez Y, Dauphin A, Carriço R, Frias JP, Vandeperre F, Otero V, Santos MR, Martins HR, Bolten AB (2017) Plastic ingestion in oceanic-stage loggerhead sea turtles (*Caretta caretta*) off the North Atlantic subtropical gyre. Marine Pollution Bulletin 121: 222-229 doi <http://dx.doi.org/10.1016/j.marpolbul.2017.06.008>

Pozo K, Gomez V, Torres M, Vera L, Nuñez D, Oyarzún P, Mendoza G, Clarke B, Fossi MC, Baini M (2019) Presence and characterization of microplastics in fish of commercial importance from the Biobío region in central Chile. Marine Pollution Bulletin 140: 315-319 doi <https://doi.org/10.1016/j.marpolbul.2019.01.025>

Renzi M, Specchiulli A, Blašković A, Manzo C, Mancinelli G, Cilenti L (2019) Marine litter in stomach content of small pelagic fishes from the Adriatic Sea: sardines (Sardina pilchardus) and anchovies (Engraulis encrasicolus). Environmental Science and Pollution Research 26: 2771-2781 doi 10.1007/s11356-018-3762-8

Rizzi M, Rodrigues FL, Medeiros L, Ortega I, Rodrigues L, Monteiro DS, Kessler F, Proietti MC (2019) Ingestion of plastic marine litter by sea turtles in southern Brazil: abundance, characteristics and potential selectivity. Marine Pollution Bulletin 140: 536-548 doi <https://doi.org/10.1016/j.marpolbul.2019.01.054>

Rummel CD, Löder MG, Fricke NF, Lang T, Griebeler E-M, Janke M, Gerdts G (2016) Plastic ingestion by pelagic and demersal fish from the North Sea and Baltic Sea. Marine Pollution Bulletin 102: 134-141 doi <https://doi.org/10.1016/j.marpolbul.2015.11.043>

Savoca S, Capillo G, Mancuso M, Bottari T, Crupi R, Branca C, Romano V, Faggio C, D’Angelo G, Spanò N (2019) Microplastics occurrence in the Tyrrhenian waters and in the gastrointestinal tract of two congener species of seabreams. Environmental Toxicology and Pharmacology 67: 35-41 doi <https://doi.org/10.1016/j.etap.2019.01.011>

Sbrana A, Valente T, Scacco U, Bianchi J, Silvestri C, Palazzo L, de Lucia GA, Valerani C, Ardizzone G, Matiddi M (2020) Spatial variability and influence of biological parameters on microplastic ingestion by Boops boops (L.) along the Italian coasts (Western Mediterranean Sea). Environmental Pollution 263: 114429 doi <https://doi.org/10.1016/j.envpol.2020.114429>

Steer M, Cole M, Thompson RC, Lindeque PK (2017) Microplastic ingestion in fish larvae in the western English Channel. Environmental Pollution 226: 250-259 doi <http://doi.org/10.1016/j.envpol.2017.03.062>

Su L, Deng H, Li B, Chen Q, Pettigrove V, Wu C, Shi H (2019) The occurrence of microplastic in specific organs in commercially caught fishes from coast and estuary area of east China. Journal of Hazardous Materials 365: 716-724 doi <https://doi.org/10.1016/j.jhazmat.2018.11.024>

Tanaka K, Takada H (2016) Microplastic fragments and microbeads in digestive tracts of planktivorous fish from urban coastal waters. Scientific Reports 6: 34351 doi <http://dx.doi.org/10.1038/srep34351>

Van Franeker JA, Bravo Rebolledo EL, Hesse E, IJsseldijk LL, Kühn S, Leopold M, Mielke L (2018) Plastic ingestion by harbour porpoises *Phocoena phocoena* in the Netherlands: Establishing a standardised method. AMBIO: A Journal of the Human Environment 47: 387-397 doi <https://doi.org/10.1007/s13280-017-1002-y>

Welden NA, Abylkhani B, Howarth LM (2018) The effects of trophic transfer and environmental factors on microplastic uptake by plaice, *Pleuronectes plastessa*, and spider crab, *Maja squinado*. Environmental Pollution 239: 351-358 doi <https://doi.org/10.1016/j.envpol.2018.03.110>

Wesch C, Barthel A-K, Braun U, Klein R, Paulus M (2016) No microplastics in benthic eelpout (*Zoarces viviparus*): An urgent need for spectroscopic analyses in microplastic detection. Environmental Research 148: 36-38 doi <http://dx.doi.org/10.1016/j.envres.2016.03.017>

Wieczorek AM, Morrison L, Croot PL, Allcock AL, MacLoughlin E, Savard O, Brownlow H, Doyle TK (2018) Frequency of microplastics in mesopelagic fishes from the Northwest Atlantic. Frontiers in Marine Science 5: 1-9 doi <http://dx.doi.org/10.3389/fmars.2018.00039>

Yamashita R, Takada H, Fukuwaka MA, Watanuki Y (2011) Physical and chemical effects of ingested plastic debris on short-tailed shearwaters, *Puffinus tenuirostris*, in the North Pacific Ocean. Marine Pollution Bulletin 62: 2845-2849 doi <http://dx.doi.org/10.1016/j.marpolbul.2011.10.008>

Zhang F, Wang X, Xu J, Zhu L, Peng G, Xu P, Li D (2019) Food-web transfer of microplastics between wild caught fish and crustaceans in East China Sea. Marine Pollution Bulletin 146: 173-182 doi <https://doi.org/10.1016/j.marpolbul.2019.05.061>

Zhu C, Li D, Sun Y, Zheng X, Peng X, Zheng K, Hu B, Luo X, Mai B (2019a) Plastic debris in marine birds from an island located in the South China Sea. Marine Pollution Bulletin 149: 110566 doi <https://doi.org/10.1016/j.marpolbul.2019.110566>

Zhu J, Yu X, Zhang Q, Li Y, Tan S, Li D, Yang Z, Wang J (2019b) Cetaceans and microplastics: First report of microplastic ingestion by a coastal delphinid, *Sousa chinensis*. Science of The Total Environment 659: 649-654 doi <https://doi.org/10.1016/j.scitotenv.2018.12.389>

Zhu L, Wang H, Chen B, Sun X, Qu K, Xia B (2019c) Microplastic ingestion in deep-sea fish from the South China Sea. Science of The Total Environment 677: 493-501 doi <https://doi.org/10.1016/j.scitotenv.2019.04.380>
